# New *In Vivo* Approach to Broaden the Thioredoxin Family Interactome in Chloroplasts

**DOI:** 10.3390/antiox11101979

**Published:** 2022-10-04

**Authors:** María Ancín, Joaquin Fernandez-Irigoyen, Enrique Santamaria, Luis Larraya, Alicia Fernández-San Millán, Jon Veramendi, Inmaculada Farran

**Affiliations:** 1 Institute for Multidisciplinary Research in Applied Biology (IMAB), Universidad Pública de Navarra (UPNA), Campus Arrosadia, 31006 Pamplona, Spain; 2 Instituto de Agrobiotecnologia (IDAB), Consejo Superior de Investigaciones Científicas (CSIC)-Gobierno de Navarra, Avenida Pamplona 123, 31192 Mutilva, Spain; 3 Proteored-ISCIII, Proteomics Unit, Navarrabiomed-Departamento de Salud-Universidad Pública de Navarra (UPNA), Campus de Ciencias de la Salud, Avda. de Barañain s/n, 31008 Pamplona, Spain; 4 IDISNA, Navarra Institute for Health Research, Avda. de Barañain s/n, 31008 Pamplona, Spain

**Keywords:** chloroplast, thioredoxin, NTRC, *Nicotiana*, proteomics, redox regulation, target proteins

## Abstract

Post-translational redox modifications provide an important mechanism for the control of major cellular processes. Thioredoxins (Trxs), which are key actors in this regulatory mechanism, are ubiquitous proteins that catalyse thiol-disulfide exchange reactions. In chloroplasts, Trx f, Trx m and NADPH-dependent Trx reductase C (NTRC) have been identified as transmitters of the redox signal by transferring electrons to downstream target enzymes. The number of characterised Trx targets has greatly increased in the last few years, but most of them were determined using in vitro procedures lacking isoform specificity. With this background, we have developed a new *in vivo* approach based on the overexpression of His-tagged single-cysteine mutants of Trx f, Trx m or NTRC into *Nicotiana benthamiana* plants. The over-expressed mutated Trxs, capable of forming a stable mixed disulfide bond with target proteins in plants, were immobilised on affinity columns packed with Ni-NTA agarose, and the covalently linked targets were eluted with dithiothreitol and identified by mass spectrometry-based proteomics. The *in vivo* approach allowed identification of 6, 9 and 42 new potential targets for Trx f, Trx m and NTRC, respectively, and an apparent specificity between NTRC and Trxs was achieved. Functional analysis showed that these targets are involved in several cellular processes.

## 1. Introduction

Plant thioredoxins (Trxs), initially identified as light-dependent regulators of key photosynthetic metabolism enzymes in chloroplasts [1], constitute a complex redox system supported by multiple Trx isoforms. In chloroplasts, several types of typical and atypical Trxs have been reported [2], with Trx f, Trx m and NTRC (C-type NADPH-dependent Trx reductase) being the most studied. The typical f-type and m-type Trxs play a key role in the ferredoxin (Fd)/Trx system of oxygenic photosynthetic organisms [3]. In this system, electrons flow from light-reduced Fd to Trxs via Fd-Trx reductase (FTR) to regulate the activity of target proteins via the reduction of specific disulfide bonds. In recent years, both types of Trx have been implicated in multiple functions, such as stress responses, starch metabolism, lipid biosynthesis, chlorophyll synthesis and breakdown, biogenesis of photosystem II (PSII), the Calvin–Benson cycle, protein folding and import or translation, and chaperone activity [4]. Conversely, the atypical Trx, NTRC, constitutes per se a redox regulatory system in chloroplasts that reduces target proteins using NADPH as the electron donor [5]. NTRC was suggested to be responsible for regulatory functions that are sometimes similar to, but in other cases distinct from, those of the classically known Fd/Trx system [6], and it serves as an efficient reductant of proteins involved in antioxidant defense, chlorophyll synthesis or gene expression [7].

Given this outlook, and because plants exhibit the most versatile Trx system, the specificity of each Trx type for its target has become fundamental for understanding Trx-controlled redox-regulated physiological processes. Before the advent of proteomics, target proteins were identified by biochemical approaches [3]. Thanks to the development of proteomics, the repertoire of Trx-targeted proteins in plants increased considerably after 2001 [8]. Two main approaches were employed for this purpose. The first method, named the reductome approach, uses thiol-specific probes to label Trx targets in a crude extract in vitro. In this procedure, the enzymatic Trx system (NADPH, NTR and Trx) is reconstituted and used to reduce disulfide bonds, thus allowing detection of Trx-targeted proteins and their identification by mass spectrometry (MS). Free sulfhydryl groups (−SH) are labelled with different probes, with cleavable isotope-coded affinity tag reagents [9] and cysteine-reactive tandem mass tags [10] being the most used due to accurate quantification of cysteine (Cys) redox status and localisation of the Trx-targeted Cys residues. The second approach is based on the covalent binding between a monocysteinic Trx mutant and its target proteins. This method takes advantage of the two-step disulfide bridge reduction reaction [11], where the more N-terminal catalytic Cys of the Trx interacts with the disulfide bridge of the target protein, reducing one Cys of the target and establishing a heterodisulfide bridge that is then reduced by the more C-terminal Cys, allowing the release of both the reduced target and the oxidised Trx. Therefore, mutation of the second Cys residue (buried Cys) into serine or alanine allows stabilisation of the heterodimer and traps potential Trx targets from different cell lysates. This strategy was performed for the first time in yeast [12], but a modification of the technique was then broadly used in plants, and consisted of mutant Trx immobilised on a resin (batch method) with Trx-bound targets eluted by adding a chemical reductant such as dithiothreitol (DTT) and identification with MS [13,14]. Moreover, some authors have compared different proteomic procedures in parallel [15,16]. An interesting point emerging from these studies is the apparent lack of specificity for Trx targets. Thus, the column-bound mutant Trx interacts with potential targets irrespective of the type used (Trx m, Trx f, Trx h or *Escherichia coli* Trx) [14]. Trying to overcome these problems, some *in vivo* studies have been performed, but given its complexity, only three approaches have been used to identify Trx targets: (i) yeast overexpressing a His-tagged-Trx h mutant variant [12], (ii) potato plants overexpressing the atypical CDSP32 in chloroplasts [17], and (iii) *Arabidopsis* plants overexpressing NTRC to capture protein complexes [18]. Considering the scarcity of *in vivo* approaches, we developed a new *in vivo* strategy based on *Agrobacterium tumefaciens*-mediated monocysteinic Trx f, Trx m or NTRC mutant overexpression in *Nicotiana benthamiana* plants, combined with MS-based proteomics. Our method led to the identification of numerous proteins that are potentially associated with plastid Trxs, allowing us to distinguish among NTRC and Trx plastidial targets *in vivo*.

## 2. Materials and Methods

### 2.1. Plant Material and Growth Conditions

*N. benthamiana* plants were grown in pots (organic soil/vermiculite, 70/30 *v*/*v*) in a phytotron with a 16-h light/8-h dark photoperiod at 28 °C under a photosynthetic photon flux density of 80 µmol m^−2^ s^−1^ (Sylvania GRO-LUX lamps, 36 W, München, Germany) and a relative humidity of 65%. Plants were watered once a week with 50% diluted Hoagland’s solution. After five weeks, fully developed leaves were used for agroinfiltration.

### 2.2. Construction of Monocysteinic Trx Mutants and Plant Transformation

Site-directed mutagenesis was performed by PCR using NtTrxf, NtTrxm [19] or AtNTRC (GenBank accession number NM129731) cDNAs as templates. The Trxf-C47S mutant was produced with C47S-f and C47S-r primers (Table S1) in order to introduce a single change to replace Cys47 with serine, and Trxf-f and Trxf-r primers (Table S1) to amplify the full gene length. The reverse primer contained a 6xHis tag. The transit peptide of the tobacco RuBisCO small subunit was amplified using Rbcs-f and Rbcsf-r primers, and translationally fused to Trxf-C47S by overlapping PCR with Rbcs-f and Trxf-r primers (Table S1). Trxm-C40S and NTRC-C457S were obtained following the same strategy using the corresponding primers (Table S1). In the case of NTRC, Cys457 corresponds to the nucleophilic Cys in the Trx domain of the protein. The chimeric genes were introduced into a pBin20 binary vector [20] under the control of the Cauliflower Mosaic Virus 35S promoter and transformed into the GV3101 *A. tumefaciens* strain.

The abaxial air spaces of fully developed leaves on 5-week-old *N. benthamiana* plants were co-infiltrated with two *A. tumefaciens* clones harbouring the single mutant constructs (Trxf-C47S, Trxm-C40S or NTRC-C457S) and the P19 gene silencing suppressor, as previously described [21], using a 1 mL syringe without a needle. A total of 40 plants were infiltrated with each construct (2 leaves per plant), as well as the negative control (a strain containing an empty pBin20 vector).

### 2.3. Protein Preparation and Purification of Target Proteins

Leaf tissue within the infiltrated area was collected 5 days post-agroinfiltration and ground immediately in liquid nitrogen. Leaves were resuspended in extraction buffer [20 mM sodium phosphate pH 7.4, 150 mM NaCl, 25 mM imidazole, 0.1% Triton X-100 and complete protease inhibitor cocktail (Roche Diagnostics, Mannheim, Germany)] and incubated for 45 min on ice. The homogenate was filtered through two layers of Miracloth (Calbiochem, Nottingham, UK), and centrifuged at 15,800× *g* for 20 min at 4 °C. The supernatant was then recovered, passed through a 0.45 µm filter and applied to affinity columns packed with Ni-NTA agarose (Qiagen, Hilden, Germany) for purification of His-tagged proteins, according to manufacturer’s instructions. The resin was washed with 20 column volumes of wash buffer (20 mM sodium phosphate pH 7.4, 150 mM NaCl, 45 mM imidazole and protease inhibitor cocktail) to fully remove non-specifically bound proteins. Target proteins were then eluted in the same wash buffer containing 20 mM DTT. Finally, monocysteinic Trxs were removed from the column using the same wash buffer supplemented with 500 mM imidazole.

### 2.4. Proteomic Analysis

#### 2.4.1. Sample Preparation

Eluates were homogenised in lysis buffer (7 M urea, 2 M thiourea, 50 mM DTT) and the protein concentration was quantified with the Bradford assay (Bio-Rad, Hercules, CA, USA) and then precipitated with a ReadyPrep 2-D cleanup kit (Bio-Rad). The protein extract for each sample was diluted in Laemmli buffer and loaded into a 1.5 mm thick polyacrylamide gel with a 4% stacking gel cast over a 15% resolving gel. The run was stopped as soon as the front entered 3 mm into the resolving gel to concentrate the whole proteome in the stacking/resolving gel interface. Bands were stained with Coomassie Brilliant Blue and excised from the gel. Protein enzymatic cleavage was carried out with trypsin (Promega, Madison, WI, USA; 1:20, *w*/*w*) at 37 °C for 16 h, as previously described [22]. Purification and concentration of peptides was performed using C18 Zip Tip Solid Phase Extraction (Millipore, Burlington, MA, USA).

#### 2.4.2. Mass Spectrometry Analysis

Peptide mixtures were separated by reverse-phase chromatography using an Eksigent nanoLC ultra 2D pump fitted with a 75 μm ID column (Eksigent 0.075 × 250). Samples were first loaded for desalting and concentration into a C18 packed precolumn (Thermo 0.5 cm length and 100 μm ID). Mobile phases were 100% water, 0.1% (*v*/*v*) formic acid (FA) (buffer A) and 100% (*v*/*v*) acetonitrile 0.1% (*v*/*v*) FA (buffer B). The column gradient was developed over 200 min as a two-step gradient: from 5% B to 25% B over 160 min and 25% B to 40% B over 21 min. The column was then equilibrated in 95% B for 8 min and 5% B for 11 min. During all processes, the precolumn was in line with the column and flow was maintained along the gradient at 300 nL/min. The peptides eluted from the column were analysed in positive ion mode using a Sciex 5600 Triple-TOF system. Data were acquired upon a survey scan performed in a mass range from 350 *m*/*z* up to 1250 *m*/*z* in a scan time of 250 ms. The top 35 peaks were selected for fragmentation. The minimum accumulation time for MS/MS was set to 100 ms, giving a total cycle time of 3.8 s. Product ions were scanned in a mass range from 230 *m*/*z* up to 1500 *m*/*z* and excluded for further fragmentation over 15 s.

#### 2.4.3. Data Analysis

The MS/MS data acquisition was performed using Analyst 1.7.1 software (Sciex, Canada) and the spectral files were processed via Protein Pilot Software v 5.0.1 (Sciex, Canada) using the Paragon™ algorithm (v 5.0.1) for database searches [23] and Progroup™ for data grouping, and were searched against the concatenated target-decoy UniProt proteome database (*Nicotiana tabacum*). The false discovery rate was determined using a non-linear fitting method [24], and the results displayed were those reporting a 1% global false discovery rate or better. Note that an *N. tabacum* database was used because it is the closest organism to *N. benthamiana* with a proteome available on UniProt.

#### 2.4.4. Peptide Quantification

Peptide quantification was performed using the Progenesis LC−MS software (ver. 2.0.5556.29015, Nonlinear Dynamics, Quayside, UK). Runs were aligned to compensate for between-run variations in our nanoLC separation system using the accurate mass measurements from full survey scans in the TOF detector and the observed retention times. To this end, all runs were aligned to a reference run automatically chosen by the software, and a master list of features considering *m*/*z* values and retention times was generated. The quality of these alignments was manually supervised with the help of quality scores provided by the software. The peptide identifications were exported from Protein Pilot software and imported into Progenesis LC−MS software, where they were matched to the respective features. Output data files were managed for subsequent statistical analyses and representation. Proteins identified by site (identification based only on a modification), reverse proteins (identified by a decoy database) and potential contaminants were filtered out. Proteins quantified with at least two unique peptides, a p-value lower than 0.05 and a Log2(fold change) > 1.8 relative to those identified in extracts from *N. benthamiana* control plants were considered as potential interacting partners of the Trx f, Trx m or NTRC proteins.

#### 2.4.5. Bioinformatics and Annotations

For this purpose, a homology search was first performed for all the identified sequences with blastp at the National Center for Biotechnology Information (https://blast.ncbi.nlm.nih.gov/Blast.cgi?PAGE=Proteins; accessed on 11 January 2021) against the nr database. In this way, *Arabidopsis* (*Arabidopsis thaliana*) orthologs were identified in order to facilitate the following information-gathering process. To determine the functional properties of the identified proteins, the protein sequences were then mapped with Gene Ontology Terms (http://geneontology.org/; accessed on 18 January 2021). Sequence alignments were carried out by the ClustalW method using the EMBL server (https://www.ebi.ac.uk/Tools/msa/clustalo/; accessed on 18 January 2021) with the default settings.

## 3. Results

### 3.1. Identification of Proteins Captured as Redox Trx Interactors

**Figure 1:**
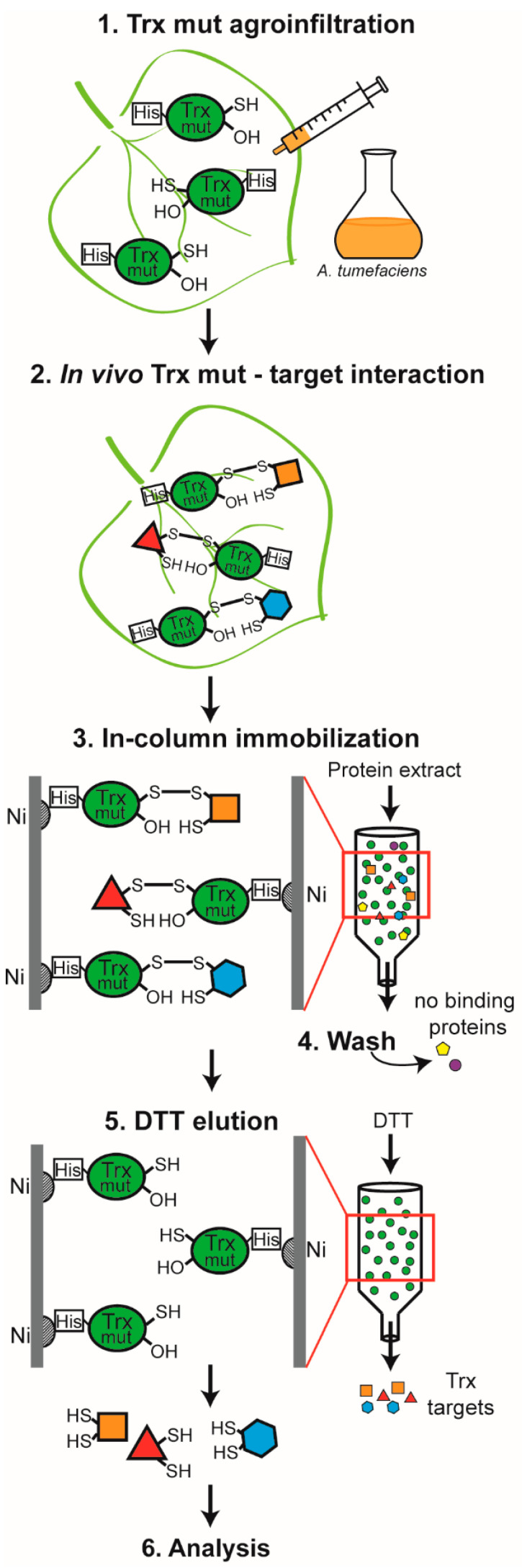
Procedure for isolation of *in vivo* Trx interactors. His-tagged single-cysteine mutants of Trx f, Trx m and NTRC were separately agroinfiltrated into *N. benthamiana* plants (1 and 2). After 5 days, leaf protein extraction was performed and the Trxs, along with their interactors, were captured in a column packed with Ni-NTA agarose (3). Finally, redox interactors were eluted with DTT (5). Trx mut: thioredoxin mutant; DTT: dithiothreitol.

To increase the number and specificity of proteins targeted by Trx f, Trx m and NTRC, we developed an *in vivo* approach (Figure 1) based on the ability of monocysteinic Trxs to form a covalent disulfide-bonded heterodimer with its targets [25]. To this end, we generated three monocysteinic His-tagged Trx mutants (Trxf-C47S, Trxm-C40S and NTRC-C457S) that were overexpressed in *N. benthamiana* plants by agroinfiltration. The Trx-targeted heterodimers covalently bound inside the chloroplast were purified by Ni affinity chromatography from leaf protein extracts 5 days after infiltration, when the expression of the mutant variants in leaf tissues was higher. Plants agroinfiltrated with a strain containing the empty pBin20 vector were used as a negative control, considering that their eluates enclose proteins that interact non-specifically with the isolation system used. Trapped targets were recovered by DTT elution and analysed by MS. Then, the columns were eluted with imidazole in order to release and visualise resin-bound Trxs (Figure S1), confirming that protein overexpression had been achieved in the agroinfiltrated plants.

**Figure 2:**
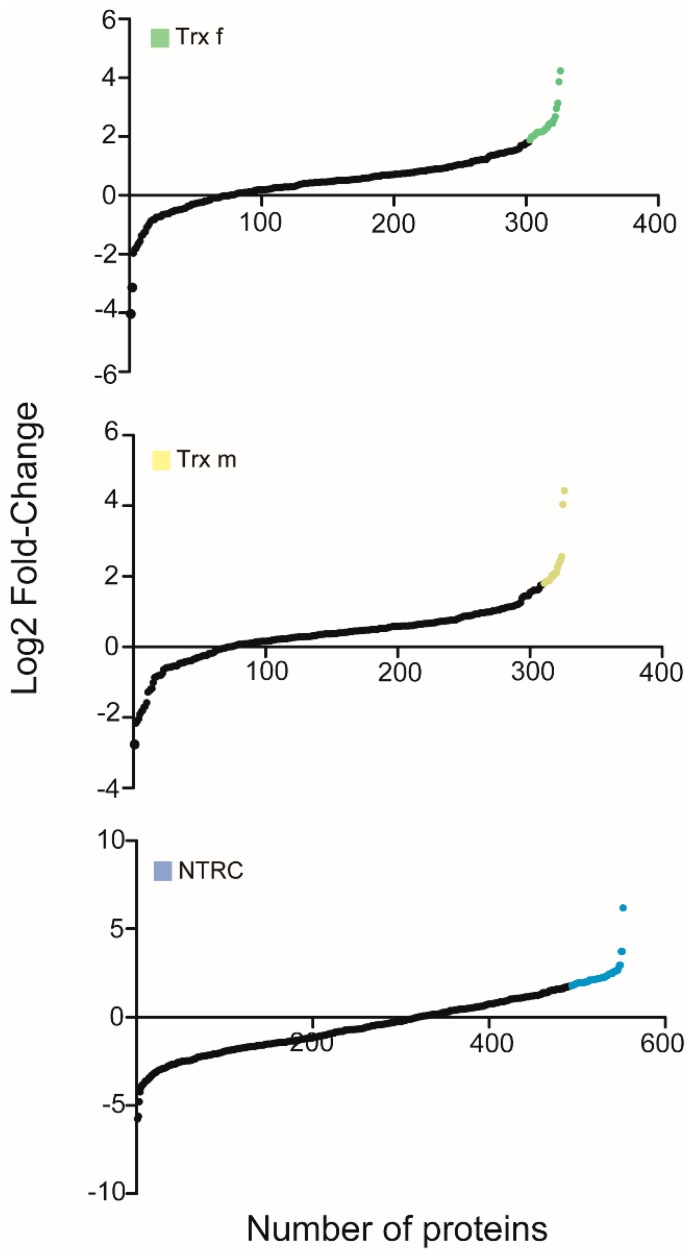
Proteomic analysis of the Trx-interacting proteins. Using quantitative mass spectrometry, eluted Trxs proteomes were compared to the control (empty-vector proteome). The candidate proteins are separately shown for Trx f (green), Trx m (yellow) and NTRC (blue).

This procedure allowed identification of hundreds of proteins in the *N. tabacum* database (Figure 2). Among them, candidate targets were selected on the basis of a Log2(fold change) > 1.8 relative to those identified in extracts from *N. benthamiana* plants agroinfiltrated with an empty vector. This selection led to the identification of 39, 41 and 120 proteins for Trx f, Trx m and NTRC, respectively, in the *N. tabacum* database (Tables S2–S4).

Although genome assembly for *N. tabacum* is being improved, *Arabidopsis* remains the best annotated plant [26]. Therefore, the predicted proteins were matched to their closest *Arabidopsis* orthologs by a blastp search to assess their likely subcellular location. This analysis showed the dominance of plastid proteins over other suborganellar locations, considering that 48, 58 and 61% of the candidate proteins for Trx f, Trx m and NTRC, respectively, were plastid-localised (Tables S2–S4). The rest of the proteins were discarded as plastid Trx target candidates. It is likely that the identification of extraplastidial proteins was a consequence of using crude leaf extracts for the purification assay, which allows interaction of non-plastidic proteins with monocysteinic Trx mutants during the extraction process.

### 3.2. Analysis of Candidate Trx Partners

Table 1 and Table 2 summarise the plastid-localised proteins identified as potential targets for Trx f (15 proteins), Trx m (18 proteins) and NTRC (68 proteins). These tables also provide information about the location of target proteins inside the chloroplast, distinguishing between stroma, thylakoid membrane or lumen. It should be noted that some of the identified proteins are well-known Trx targets, such as NADP-glyceraldehyde-3-phosphate dehydrogenase (NADP-GAPDH), sedoheptulose-bisphosphatase (SBPase) or RuBisCO activase [27], confirming the applicability of the approach. Since Trx-targeted proteins often form an inter- or intramolecular Trx-reducible disulfide, we further analysed the number of conserved Cys residues in the new potential Trx partners by ClustalW amino acid sequence alignment (Table 1 and Table 2). We found six previously undescribed putative targets for Trx f and m: Trx-like 4, phosphoglucan water dikinase (PWD), amidophosphoribosyltransferase 2 (ATase2), 30S ribosomal protein S3, uridine kinase and cis-abienol synthase; and three more only for Trx m: Trx-like 2, starch synthase and 50S ribosomal protein L18 (Table 1; bold and Table S5).

Concerning NTRC, we found six proteins that were already described as NTRC partners: 2-Cys peroxiredoxin (Prx) B, Trx m, FTR, ferredoxin-2, fructose-1,6-bisphosphatase (FBPase) and the glucose-1-phosphate adenylyltransferase (AGPase) small subunit. However, we also identified 42 new targets for NTRC: 28 of them have already been described as targets of other Trx types, but not for NTRC (Table 2; underlined), while the other 14 are completely new (Table 2; bold and Table S5). It should be mentioned that 20 identified proteins had to be discarded as redox-interacting proteins (Table 2; grey) because of their lumenal localisation or the lack of conserved Cys.

**Figure 3:**
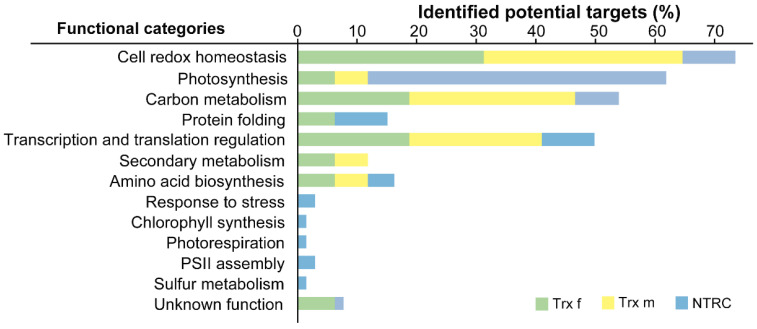
Functional classification of proteins identified as putative Trx f, Trx m or NTRC partners in chloroplasts. The plot represents the percentage of total targets for each Trx in each functional category.

When the identified targets were grouped into different chloroplast processes, we found that they were involved in diverse biological functions (Figure 3). Trx f and Trx m targets were placed mostly into cell redox homeostasis (up to 30%) and carbon metabolism (~19 and 28%, respectively). However, the main represented group for NTRC was photosynthesis (~50%), followed by cell redox homeostasis, protein folding and transcription and translation regulation (~9% each one).

**Figure 4:**
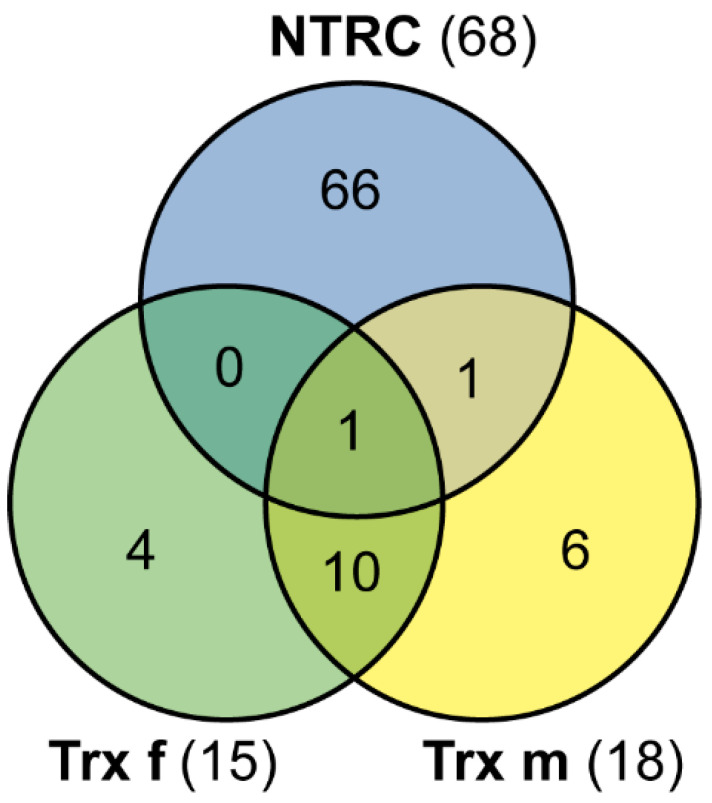
Comparison of Trx specificity. Venn diagram representation showing the overlap between candidate target proteins for Trx f, Trx m and NTRC.

Analysing the specificity among different Trxs in our system, we observed that plastid-localised targets associated with Trx f and Trx m were mainly coincident (Table 1). Among the selected candidate targets, 11 proteins were shared between Trx f and Trx m (Figure 4 and Table 1), which implies that the specificity between these Trxs and their targets is poorly conserved according to this *in vivo* approach. In contrast, NTRC seems to have better conserved the specificity of its targets, considering that only two proteins were also identified as Trx f and/or m targets in this assay (Figure 4). These proteins were 2-Cys Prx, which was captured as a partner of the three Trxs, and glutathione peroxidase, which is listed as a Trx m and NTRC target protein (Table 1 and Table 2).

## 4. Discussion

For decades, numerous approaches have contributed to increasing the knowledge of plant Trx-interacting proteins, not only in chloroplasts, but also in other compartments [8,10]. Among the chloroplast thioredoxins, Trx f and m targets have been more frequent subjects of study than NTRC targets. In the latter case, only two previous works have identified putative targets of its Trx domain [6,18]. In general, almost all of these studies have been performed in vitro, which leads to some limitations, such as the lack of isoform specificity. Against this background, we developed a new *in vivo* strategy to explore Trx f, Trx m and NTRC interactomes that complements previous studies and, at the same time, gives insights into thioredoxin type specificity.

### 4.1. Novelty and Specificity of the Approach

Unlike the in vitro mutant Trx affinity trapping-based methods, here we performed an alternative approach, where Trxs-targeted protein interactions were stabilised *in vivo* inside plant chloroplasts. Infiltration of *A. tumefaciens* into *N. benthamiana* leaves is frequently used to facilitate mass production of valuable proteins, a procedure known as molecular farming [28]. In this work, we used this technique to overexpress a monocysteinic His-tagged Trx mutant that was targeted in the chloroplast by means of the RuBisCO small subunit transit peptide. Inside the chloroplast, this mutant variant formed a stable heterodimer with its targets, which were finally trapped via affinity purification. This approach allowed the *in vivo* identification of 15, 18 and 68 potential targets for Trx f, Trx m and NTRC, respectively (Table 1 and Table 2). A similar approach has been broadly used to identify protein–protein interactions in mammalian cells. In such applications, cells are transfected with a plasmid coding for a tagged-bait protein that is then isolated, together with bound proteins, using a specific chemical or biological ligand linked to a solid support [29]. However, this technique has not been used to trap redox interactors. Indeed, only two *in vivo* approaches have been used to identify Trx targets in plants [17,18]. Therefore, our approach represents the first attempt to identify Trx f, Trx m and NTRC redox targets *in vivo*.

Regarding the specificity of this *in vivo* approach against Trx partners, our results show low specificity between the classical f and m types of Trxs, although it was preserved in the case of NTRC (Figure 4). The lack of Trx-type specificity is common to both the affinity column and reductome approaches, as was appreciated in a previous work in which targets trapped on mutant Trx f and m columns were compared [14]. This was partially explained by assuming that the replacement of one Cys in the active site by serine causes a slight change in the microenvironment of the protein, which abolishes specificity. This view could also be applied in our study, where such interaction occurred *in vivo*. Similarly, it has been reported [30] that the mutant Trx m protein traps several chloroplast targets that are known to prefer Trx f, which itself, when mutated, was ineffective in binding such target enzymes [30]. Moreover, some well-known Trx targets have also been trapped by mutant forms of Trx-like proteins such as CDSP32 or HCF164 [17,31], or even glutaredoxins (Grx) [32]. However, our results show a gain in NTRC specificity for its targets (Figure 4), which could be explained by important differences between classical Trxs and NTRC with regard to protein structure. While Trx f- and Trx m-identified targets share the same main processes (cell redox homeostasis, carbon metabolism or transcription and translation regulation), NTRC targets are mainly involved in photosynthesis. Moreover, there are processes that can be exclusively assigned to Trx f or m (secondary metabolism) or NTRC (response to stress, chlorophyll synthesis, photorespiration, PSII assembly or sulfur metabolism) (Figure 3). This agrees with a regulatory role for NTRC in the chloroplast redox network that is distinct from the FTR/Trx system [6,33], although their activities seem to be interconnected [34,35]. In fact, there is one study where several targets identified by affinity chromatography using an in vitro approach showed distinct interaction efficiencies with NTRC and Trx f [6].

### 4.2. New Potential Trx f, Trx m and NTRC Target Proteins

This study led to the identification of 102 proteins linked to Trx f, Trx m and NTRC (Table 1 and Table 2). Many of them were previously identified as potential Trx targets by distinct methodologies. However, 23 of them were completely newly identified partners that fulfilled two requirements: (i) harbouring conserved Cys residues (Table S5); and (ii) location in the stroma, or at least possessing conserved Cys residues on the stromal side of the thylakoid membrane. Trapped proteins with no conserved Cys residues were considered components of complexes that could be eluted when linked to Trx targets. These types of proteins, such as superoxide dismutase [Fe]2, PSI reaction center subunit IV A or B, PSII 22 kDa protein, cytochrome (Cyt) b559 subunit alpha or peptidyl-prolyl cis-trans isomerase FKBP19 (Table 1 and Table 2), are not recognised as putative redox interactors. On the other hand, we have also identified some lumenally located proteins such as the immunophilins FKBP19, FKBP16-3, CYP20-2 and CYP38 or the 29 kDa thylakoid lumenal proteins TL29 or TL20.3 (Table 1 and Table 2), none of which were considered as potential targets in this study. Below, we briefly discuss only the feasible targets identified with this approach.

#### 4.2.1. Trx f and Trx m

*Cell antioxidants and redox homeostasis*. Two atypical Trxs belonging to the Lilium or ACHT family (Lilium 2 and 5) were identified as Trx f and m partners for the first time in this study (Table 1). Although Lilium 2 was only shown to be efficiently reduced by glutathione [36], our results indicate that Trx f and/or m could be direct reductants of this family (two conserved Cys residues were found for each protein). The following proteins found in this category were already identified as Trx targets. First, two Prxs localised in the stroma (Table 1), the typical 2-Cys Prx B and the atypical Prx IIE [15,16,17,30,37], which are involved in antioxidant defense and redox signalling. Both of them are known to be reduced in vitro by typical and atypical Trxs or Grxs [38,39,40,41]. Then, peptide methionine sulfoxide reductase (MSRA4) [15,42], which acts against oxidative damage, was identified as a putative Trx m target (Table 1). Accordingly, tobacco plants overexpressing Trx m in chloroplasts specifically displayed increased MSR capacity [43]. Finally, another enzyme with a role in protecting cells against oxidative damage, PHGPx, was detected as a Trx m partner (Table 1). Both y- and z-type Trxs were demonstrated to reduce PHGPx in vitro efficiently [44,45], although only mitochondrial PHGPx has been identified as a putative Trx interactor [13].

*Photosynthesis*. A single protein involved in photosynthetic light reactions was identified as a Trx target in this assay, PGR5-like protein (PGRL1) (Table 1), which participates in the cyclic electron flow around PSI in chloroplasts. Although it has not been identified previously as a Trx target by proteomic approaches, an in vitro interaction between PGRL1 and Trx m, and less efficiently with Trx f, has been demonstrated [46]. Moreover, in vitro experiments have revealed an inhibitory effect of *Arabidopsis* Trx m4 on the PGR-dependent pathway, as well as an inhibition of this pathway in tobacco plants overexpressing Trx m *in vivo* [47], pointing to Trx m as the principal regulator of PGR-dependent electron flow.

*Carbon metabolism*. We found two new putative target enzymes that participate in starch metabolism, a starch synthase (SS) (identified as a granule-bound SS (GBSS1) according to its homology with *Arabidopsis* sequences) and PWD, which catalyses the phosphorylation of starch required for degradation. There is evidence indicating a redox regulation of *Arabidopsis* soluble SS1 and SS3 [48], and the SS1 isoform is known to be activated in vitro by Trx f1, Trx m4 and NTRC [49]. However, there is no evidence to date relating GBSSs to Trx modulation, but sequence alignment among different species led to our identification of six conserved Cys residues (Table 1). Similar to AtSS1, it cannot be discarded that some of these residues might affect both the activity and redox sensitivity of the enzyme. In the case of PWD, our work constitutes the first evidence of its regulation by both Trx f and m (seven conserved Cys residues (Table 1)).

In addition, other proteins were also identified as Trx targets, but not for the first time. Chloroplastic malate dehydrogenase (NADP-MDH), which functions in the malate valve to export excess reductive power from the chloroplasts, was identified as a Trx m partner (Table 1). It is considered a classical Trx target [27] that could be activated by different Trxs in vitro and *in vivo* [6,39,50,51,52], indicating that Trx f/m specificity has not been completely addressed. Moreover, two Calvin–Benson cycle enzymes, NADP-GAPDH subunit B and SBPase, were identified (Table 1), also known as classical Trx target proteins [15,30,42] that have been reported to be regulated in vitro by Trx f [6,50], but regulated *in vivo* by Trx m [51]. Finally, RuBisCO activase, an enzyme that regulates the activity of the entire Calvin–Benson cycle via regulation of RuBisCO, was identified as a Trx f and m target (Table 1). It was previously reported to be specifically activated by Trx f [53], and it was subsequently identified as a Trx target by different proteomic approaches [15,30,42].

*Transcription and translation regulation*. Four proteins related to this function were identified as new potential targets for Trx f and m. Two of them were translation-related proteins: 50 s ribosomal protein L18 and 30 s ribosomal protein S3 (Table 1). Although these proteins are generally not considered to undergo Cys oxidoreduction, in a previous study, a large number of ribosomal proteins were identified as potential Trx targets [9]. There is further evidence about the role of Trxs in protein translation, such as the known capability of light to activate translation [54] and to stabilise mRNA [55]. We found that ribosomal proteins L18 and S3 showed one and three conserved Cys residues, respectively (Table 1), suggesting that Trxs could redox modulate the formation of ribosomal complexes, as occurs with the GAPDH/PRK/CP12 complex [56]. Another two enzymes involved in nucleotide metabolism were found as putative Trx f and m targets: uridine kinase-like protein 1 and ATase2 (Table 1). In both cases, no evidence about a possible redox regulation has been reported in the literature to date. In the ATase2, sequence alignment indicated the presence of nine conserved Cys residues (Table 1).

*Secondary metabolism*. An enzyme involved in Z-abienol biosynthesis was also identified as a new Trx putative target (Table 1). Cis-abienol synthase participates in the biosynthesis of this diterpene, which is a precursor of important flavours and aromas in tobacco glandular trichomes. Due to its specificity, it is not possible to perform an alignment to permit the assignment of conserved Cys, but the tobacco sequence contains 20 Cys residues.

*Amino acid biosynthesis*. A protein involved in the metabolism of amino acids that belongs to the 5′-adenylylsulfate reductase family, APR 2, was identified. This key enzyme in the plant sulfate assimilation pathway contains a Trx-like domain, and pre-incubations with high concentrations of DTT or Trx m lead to inactivation of the enzyme [57]. Together with our results, this indicates a putative interaction with Trxs.

#### 4.2.2. NTRC

*Cell antioxidants and redox homeostasis*. Six proteins related to this function were identified as putative NTRC targets (Table 2). The enzyme 2-Cys Prx B, also identified as a putative Trx f and m target (Table 1), is an established NTRC target that has been identified as the primary electron donor for 2-Cys Prx *in vivo* [6,33,58]. It has also been shown that the redox balance of 2-Cys Prx is linked to both NTRC and the Fd/Trx system [34,35], confirming our results and validating the experimental approach. Subsequently, we found the CBSX1, DHAR3, tAPX and PHGPx proteins as potential NTRC targets (Table 2). All of them were previously shown to interact or be reduced by Trxs [15,42,45,59], but no direct relationship to NTRC has been shown to date. Finally, another new potential NTRC target was identified, Trx m4 (Table 2). Supporting this finding, an *in vivo* interaction between NTRC and other Trx m isoforms has been demonstrated via a bimolecular fluorescence complementation test [33], although in vitro experiments showed that NTRC could not reduce Trx m [6].

*Photosynthesis*. Our approach enabled the identification of 25 putative partners involved in photosynthetic light reactions (Table 2). Among the six identified PSI core subunits, the N subunit was previously reported as a disulfide-containing protein in *Arabidopsis* [60] and as a Trx target in thylakoid membranes [61]. The remaining proteins, subunits A, B, C, D2 and L could also be considered as new putative targets of NTRC, with an emphasis on PSI-C, which harbours nine conserved Cys residues (Table 2) and is located on the stromal side of the thylakoid membrane. The identification of so many subunits could be a result of PSI complex capture, as previously reported for NTRC and some NDH complex subunits [62]. PSII core proteins (D1, D2, CP43 and CP47) were also identified as new potential NTRC partners (Table 2), although a direct interaction with Trx m in assisting the biogenesis of PSII has been reported [63]. In the same way, four chlorophyll a/b binding proteins were newly identified for NTRC (Table 2), although they were previously identified as Trx targets in thylakoid membranes [61]. Two oxygen-evolving enhancer proteins were also identified as NTRC targets (Table 2), and these were previously described as putative Trx partners in proteomic approaches [42,61]. Assuming that these proteins are located in the thylakoid lumen associated with the PSII complex, we postulated that they also could have been trapped as part of this complex.

This work further identified three components of the Cytb6f complex (Cyt b6, Cyt b6-f complex iron sulfur subunit and Cyt f) as new potential NTRC partners for the first time, and they have two, four and two conserved Cys residues, respectively (Table 2). Fd and FTR were also identified (Table 2), but both have already been associated with NTRC [18,33], suggesting a link between NTRC and the FTR–Trx system. Finally, four subunits of the ATP synthase were found (Table 2), but the α, β and δ subunits of CF1 were previously identified as putative Trx partners by proteomic approaches [31,61]. The remaining subunit (ATP synthase subunit b) has never been identified as a Trx target, so it could be considered a new putative target with one conserved Cys (Table 2).

*Carbon metabolism*. Among proteins involved in carbon metabolism, four Calvin–Benson cycle enzymes were identified (Table 2), including FBPase, which could be considered an already-known NTRC interactor, given its role in FBPase regulation *in vivo* [33]. The other three enzymes, the RuBisCO large subunit, triosephosphate isomerase and ribose-5-phosphate isomerase, were recognised as new potential targets for NTRC in this study, although they were identified as partners for other Trxs in earlier proteomic approaches [13,15,21,30,42,61]. The other enzyme detected in this group was the small subunit of AGPase, which is considered a key enzyme in starch synthesis. A redox activation of the enzyme exerted by NTRC has been demonstrated both in vitro and *in vivo* [64,65], and a direct interaction between both proteins was also confirmed by yeast two-hybrid analysis [66]. Such evidence, together with our results, strongly indicates a role for NTRC in AGPase redox modulation.

*Protein folding*. Two chaperonins involved in protein folding were identified as NTRC putative targets (Table 2). The first of these is the β subunit of the 60 kDa chaperonin (CPN60), which was previously identified as a potential Trx target candidate [67,68,69]. The second one, CPN10, is a co-chaperonin of CPN60 that was identified in this study as a new putative target for Trx or NTRC and has two conserved Cys residues.

*Transcription and translation regulation*. RNA-binding protein CP29B and chloroplast stem-loop-binding protein of 41 kDa b have been defined before as potential Trx targets via proteomic approaches [15], but no direct interaction with NTRC has been shown to date. Pentatricopeptide repeat-containing protein At4g30825 and nucleoid-associated protein At4g30620 have never been associated with putative redox modulation, therefore they can be considered new potential targets for both Trxs and NTRC due to the presence of nine and one conserved Cys residues, respectively (Table 2).

*Amino acid biosynthesis*/*Nitrogen assimilation*. We detected Fd-dependent glutamate synthase 1, which participates in glutamate synthesis and photorespiration, and this protein has been reported to be activated by DTT and reduced Trxs, more efficiently by Trx m [70]. Additionally, it has been observed as a potential Trx target in amyloplasts [71]. Ketol-acid reductoisomerase, which is involved in valine and isoleucine biosynthesis, and ornithine carbamoyltransferase, implicated in the synthesis of arginine, were previously reported as potential Trx targets in cereal and medicago seeds [16,68], as well as amyloplasts [71]. However, this is the first time where an interaction with NTRC can be reported for all of these proteins.

*Sulfur metabolism.* Cysteine synthase was identified as an NTRC target in this approach (Table 2). It has been determined that this enzyme requires DTT for activity [72] and it was identified as a Trx target [14], but no evidence about a putative interaction with NTRC has been previously shown.

*Other processes*. Two enzymes involved in the tetrapyrrole pathway (CPOX) and photorespiration (phosphoglycolate phosphatase 1B) were identified as new putative NTRC targets (Table 2). There is no previous evidence of a redox regulation of these enzymes; however, they contain two and four conserved Cys residues, respectively (Table 2).

## 5. Conclusions

In summary, our new strategy based on *in vivo* trapping of Trx interactors in *N. benthamiana* chloroplasts using monocysteinic mutant forms of Trx f, Trx m and NTRC led to the identification of six and nine new potential target proteins for Trxs f and Trx m, respectively, and 14 for NTRC. Despite the apparent lack of specificity shown for Trx f and Trx m, a significant specificity for NTRC was observed. The newly identified proteins contain conserved Cys residues, are located in the stroma or in the thylakoid membrane (although the putative stromal location of the conserved Cys residues remains to be analysed) and they function across a spectrum of processes.

## Figures and Tables

**Table 1:** List of chloroplast proteins identified as potential Trx f and Trx m targets by *in vivo* interaction with the monocysteinic mutant through agroinfiltration of *N. benthamiana* plants. The table shows identified proteins in an *N. tabacum* database, as well as their orthologs in *Arabidopsis*, with the corresponding identity %. The specificity for each isoform and the corresponding subcellular localisation inside the chloroplast are indicated. The potential new targets are indicated in bold and the number of conserved Cys residues is shown. Thylakoid m: thylakoid membrane.

*Nicotiana tabacum*		*Arabidopsis thaliana*			Trx f	Trx m	Cys	Subcellular Localisation
UniProtKB	Protein Name	UniProtKB	Protein Name	Identity %				
*Cell antioxidant and redox homeostasis*								
A0A1S4A3V7	2-Cys peroxiredoxin BAS1	Q9C5R8	2-Cys peroxiredoxin BAS1-like(2-Cys Prx B)	77	x	x		stroma
A0A1S4A969	Glutathione peroxidase	P52032	Phospholipid hydroperoxide glutathione peroxidase 1 (PHGPx)	72.4		x		stroma
A0A1S4C620	Peptide methionine sulfoxide reductase-like	P54150	Peptide methionine sulfoxide reductase A4 (MSRA4)	62	x	x		stroma
A0A1S4B900	Peroxiredoxin Q	Q9LU86	Peroxiredoxin Q (Prx Q)	76.3	x	x		lumen
A0A1S4D678	Peroxiredoxin-2E-2	Q949U7	Peroxiredoxin-2E (Prx IIE)	57.7	x			stroma
**A0A1S4ASD9**	**Thioredoxin-like 2**	**Q8LCT3-2**	**Thioredoxin-like 2-2 (Lilium 2)**	**65.5**		**x**	**2**	**stroma**
**A0A1S4D2Y9**	**Thioredoxin-like 4**	**Q9C5C5**	**Thioredoxin-like 4 (Lilium 5)**	**66.3**	**x**	**x**	**2**	**stroma**
*Photosynthesis*								
A0A1S4CIH3	PGR5-like protein 1A	Q8H112	PGR5-like protein 1A (PGRL1)	72.8	x	x		thylakoid m
*Carbon metabolism*								
A0A1S4ATB8	Glyceraldehyde-3-phosphate dehydrogenase	P25857	Glyceraldehyde-3-phosphate dehydrogenase GAPB (NADP-GAPDH)	85.8	x			stroma
A0A1S3YQS9	Malate dehydrogenase (NADP)	Q8H1E2	Malate dehydrogenase (NADP-MDH)	79.4		x		stroma
**A0A1S4CZ71**	**Phosphoglucan water dikinase**	**Q6ZY51**	**Phosphoglucan water dikinase (PWD)**	**61.2**	**x**	**x**	**7**	**stroma**
A0A1S4A3L9	Ribulose bisphosphate carboxylase/oxygenase activase 1	P10896	Ribulose bisphosphate carboxylase/oxygenase activase (RuBisCO activase)	78.6	x	x		stroma
A0A1S3YEX0	Sedoheptulose-1,7-bisphosphatase	P46283	Sedoheptulose-1,7-bisphosphatase (SBPase)	81.5		x		stroma
**A0A1S4DEY3**	**Starch synthase**	**Q9MAQ0**	**Granule-bound starch synthase 1 (GBSS1)**	**74.1**		**x**	**6**	**stroma**
*Protein folding*								
A0A1S4DGW7	Peptidylprolyl isomerase	Q9LYR5	Peptidyl-prolyl cis-trans isomerase FKBP19 (PPIase FKBP19)	87.7	x			lumen
*Transcription and translation regulation*								
**A0A1S4DL03**	**50S ribosomal protein L18**	**Q9SX68**	**50S ribosomal protein L18**	**83.8**		**x**	**1**	**stroma**
**Q6T7F3**	**Amidophosphoribosyltransferase**	**Q9STG9**	**Amidophosphoribosyltransferase 2 (ATase2)**	**77.6**	**x**	**x**	**9**	**stroma**
**A0A140G1V5**	**Ribosomal protein S3**	**P56798**	**30S ribosomal protein S3**	**87.6**	**x**	**x**	**3**	**stroma**
**A0A1S4CR05**	**Uridine kinase**	**Q9FKS0**	**Uridine kinase-like protein 1 (UK)**	**87.4**	**x**	**x**	**#**	**stroma**
*Secondary metabolism*								
A0A1S4B8Q6	Cis-abienol synthase	G3CCC1	Cis-abienol synthase (ABS) *		x	x	#	stroma
*Amino acid biosynthesis*								
A0A1S4DHJ8	5′-adenylylsulfate reductase 2	P92981	5′-adenylylsulfate reductase 2 (APR2)	75.4	x	x		stroma
*Unknown function*								
A0A1S4CQQ3	Thylakoid lumenal 29 kDa protein	P82281	Thylakoid lumenal 29 kDa protein (TL29)	69.4	x			lumen

* No homologues are present in the *Arabidopsis* genome. # The database is not sufficiently developed to permit the assignment of conserved amino acids.

**Table 2:** List of chloroplast proteins identified as potential NTRC targets by *in vivo* interaction with the monocysteinic mutant through agroinfiltration of *N. benthamiana* plants. The table shows identified proteins in an *N. tabacum* database, as well as their orthologs in *Arabidopsis*, with the corresponding identity %. Localisation inside the chloroplast is indicated. The new potential targets for any Trx type are indicated in bold and the number of conserved Cys residues is shown. The proteins previously identified as targets of other Trx types, except for NTRC, are underlined. The proteins located in the lumen or without conserved Cys residues are indicated in grey text. Thylakoid m: thylakoid membrane; Thylakoid ls: thylakoid lumenal side.

*Nicotiana tabacum*		*Arabidopsis thaliana*			Cys	Subcellular Localisation
UniProtKB	Protein Name	UniProtKB	Protein Name	Identity %		
*Cell antioxidant and redox homeostasis*						
A0A1S4A3V7	2-Cys peroxiredoxin BAS1	Q9C5R8	2-Cys peroxiredoxin BAS1-like (2-Cys Prx B)	77		stroma
A0A1S4B1Q8	CBS domain-containing protein CBSX1	O23193	CBS domain-containing protein CBSX1 (CDCP2)	63.6		stroma
A0A1S3Y8V8	Glutathione S-transferase DHAR3	Q8LE52	GSH-dependent dehydroascorbate reductase 3 (DHAR3)	72.5		stroma
A0A1S3ZZS2	Probable L-ascorbate peroxidase 6	Q42593	L-ascorbate peroxidase T (tAPX)	73.7		thylakoid m
A0A1S4A969	Glutathione peroxidase	P52032	Phospholipid hydroperoxide glutathione peroxidase 1 (PHGPx)	72.4		stroma
W0KRH1	Superoxide dismutase	Q9LU64	Superoxide dismutase [Fe] 2 (FSD2)	59.1	0	thylakoid m
A0A1S4CCB3	Thioredoxin-like	Q9SEU6	Thioredoxin M4 (Trx m4)	53.3		stroma
*Photosynthesis*						
**A0A140G1P8**	**ATP synthase CF0 B subunit**	**P56759**	**ATP synthase subunit b**	**88.6**	**1**	**thylakoid m**
A0A1S4CSA5	ATP synthase delta chain	Q9SSS9	ATP synthase subunit delta	60.5		thylakoid m
P00823	ATP synthase subunit alpha	P56757	ATP synthase subunit alpha	94		thylakoid m
A0A140G1S2	ATP synthase subunit beta	P19366	ATP synthase subunit beta	93		thylakoid m
A0A1S4CBW5	Chlorophyll a-b binding protein	Q9SY97	Photosystem I chlorophyll a-b binding protein 3-1 (Lhca3.1)	89.4	0	thylakoid m
Q0PWS6	Chlorophyll a-b binding protein	Q9C639	Photosystem I chlorophyll a-b binding protein 5 (Lhca5)	38	0	thylakoid m
Q40512	Chlorophyll a-b binding protein	Q01667	Photosystem I chlorophyll a-b binding protein 6 (Lhca1)	87.4	3	thylakoid m
A0A1S4BMB0	Chlorophyll a-b binding protein	Q9SHR7	Photosystem II chlorophyll a-b binding protein 2.1 (Lhcb2.1)	89		thylakoid m
A0A1S4DIE1	Chlorophyll a-b binding protein	Q9S7M0	Photosystem II chlorophyll a-b binding protein 3 (Lhcb3)	87.5		thylakoid m
Q0PWS7	Chlorophyll a-b binding protein	Q07473	Photosystem II chlorophyll a-b binding protein CP29.1 (Lhcb4.1)	86.6		thylakoid m
A0A140G1T3	Cytochrome b559 subunit alpha	P56779	Cytochrome b559 subunit alpha	99	0	thylakoid m
**A0A1S3XVT6**	**Cytochrome b6**	**P56773**	**Cytochrome b6**	**98**	**2**	**thylakoid m**
**A0A1S4B832**	**Cytochrome b6-f complex iron-sulfur subunit**	**Q9ZR03**	**Cytochrome b6-f complex iron-sulfur subunit (RISP)**	**77.8**	**4**	**thylakoid m**
**A0A140G1S8**	**Cytochrome f**	**P56771**	**Cytochrome f**	**90**	**2**	**thylakoid m**
A0A1S3YVN4	Ferredoxin	P16972	Ferredoxin-2 (Fd2)	65.7	4	stroma
A0A1S4B5N2	Ferredoxin-thioredoxin reductase	A0A1P8BDN6	Ferredoxin-thioredoxin reductase subunit A (Variable subunit) 2	46.5		stroma
Q84QE8	Oxygen evolving complex 33 kDa photosystem II protein	Q9S841	Oxygen-evolving enhancer protein 1-2 (OEE-1)	81.3		thylakoid ls
A0A1S4BMY9	Oxygen-evolving enhancer protein 2-2	Q42029	Oxygen-evolving enhancer protein 2-1 (OEE-2)	72.9		thylakoid ls
A0A1S3XRM3	Oxygen-evolving enhancer protein 3-2	Q41932	Oxygen-evolving enhancer protein 3-2 (OEE-3)	68.8	0	thylakoid ls
**A0A140G1 × 0**	**Photosystem I iron-sulfur center**	**P62090**	**Photosystem I iron-sulfur center (PSI-C)**	**100**	**9**	**thylakoid m**
**A0A140G1R3**	**Photosystem I P700 chlorophyll a apoprotein A1**	**P56766**	**Photosystem I P700 chlorophyll a apoprotein A1 (PSI-A)**	**98**	**4**	**thylakoid m**
**A0A140G1R2**	**Photosystem I P700 chlorophyll a apoprotein A2**	**P56767**	**Photosystem I P700 chlorophyll a apoprotein A2 (PSI-B)**	**98**	**2**	**thylakoid m**
**A0A1S3ZIE1**	**Photosystem I reaction center subunit II**	**Q9SA56**	**Photosystem I reaction center subunit II-2 (PSI-D2)**	**76.6**	**1**	**thylakoid m**
A0A1S4CFV4	Photosystem I reaction center subunit IV A	Q9S831	Photosystem I reaction center subunit IV A (PSI-E1)		0	thylakoid m
A0A1S4CYN6	Photosystem I reaction center subunit IV B	Q9S714	Photosystem I reaction center subunit IV B (PSI-E2)	58.4	0	thylakoid m
D2K7Z2	Photosystem I reaction center subunit	P49107	Photosystem I reaction center subunit N (PSI-N)	69.1		thylakoid m
A0A1S4CR54	Photosystem I reaction center subunit VI-1	Q9SUI7	Photosystem I reaction center subunit VI-1 (PSI-H1)	77.2	0	thylakoid m
**A0A1S4BQS3**	**Photosystem I reaction center subunit XI**	**Q9SUI4**	**Photosystem I reaction center subunit XI (PSI-L)**	**80.7**	**1**	**thylakoid m**
A0A1S3YQ87	Photosystem II 22 kDa protein	Q9XF91	Photosystem II 22 kDa protein (CP22)	73.2	0	thylakoid m
A0A140G1Q8	Photosystem II CP43 reaction center protein	P56778	Photosystem II CP43 reaction center protein	98	3	thylakoid m
A0A140G1U3	Photosystem II CP47 reaction center protein	P56777	Photosystem II CP47 reaction center protein	98.6		thylakoid m
A0A140G1Q7	Photosystem II D2 protein	P56761	Photosystem II D2 protein	99		thylakoid m
A0A140G1P2	Photosystem II D1 protein	P83755	Photosystem II D1 protein	99.7		thylakoid m
A0A1S4A1K3	Plastocyanin	P42699	Plastocyanin major isoform	67.7	0	thylakoid ls
*Carbon metabolism*						
A0A1S4A023	Fructose-1,6-bisphosphatase	P25851	Fructose-1,6-bisphosphatase 1 (FBPase 1)	86.5		stroma
A7XAQ5	Glucose-1-phosphate adenylyltransferase	P55228	Glucose-1-phosphate adenylyltransferase small subunit (AGPase B)	87.5		stroma
A0A1S3Z1 × 1	Probable ribose-5-phosphate isomerase 3	Q9S726	Probable ribose-5-phosphate isomerase 3	68.3		stroma
A0A140G1S3	Ribulose bisphosphate carboxylase large chain	O03042	Ribulose bisphosphate carboxylase large chain (RuBisCO LSU)	94		stroma
A0A1S3X2Z0	Triosephosphate isomerase	Q9SKP6	Triosephosphate isomerase (TPI)	79.4		stroma
*Protein folding*						
**A0A1S4AH01**	**10 kDa chaperonin-like**	**Q9M1C2**	**10 kDa chaperonin 1 (CPN10)**	**71**	**2**	**stroma**
A0A077DBL2	20 kDa chaperonin	O65282	20 kDa chaperonin (CPN20)	74	0	stroma
A0A1S4AWT3	Peptidyl-prolyl cis-trans isomerase	Q9ASS6	Peptidyl-prolyl cis-trans isomerase CYP20-2	69.3	0	thylakoid ls
A0A1S3ZH83	Peptidyl-prolyl cis-trans isomerase CYP38	Q9SSA5	Peptidyl-prolyl cis-trans isomerase CYP38	76.8	0	lumen
A0A1S3XJV2	Peptidyl-prolyl isomerase	O22870	Peptidyl-prolyl cis-trans isomerase FKBP16-3	65.5	0	lumen
A0A1S4DIY1	RuBisCO large subunit-binding protein subunit beta	P21240	Chaperonin 60 subunit beta 1 (CPN60)	81.8		stroma
*Transcription and translation regulation*						
A0A1S4CYJ5	29 kDa ribonucleoprotein A	Q9ZUU4	RNA-binding protein CP29B	59.9		stroma
A0A1S3XX03	31 kDa ribonucleoprotein	Q04836	31 kDa ribonucleoprotein	53	0	stroma
A0A1S3Z334	Chloroplast stem-loop binding protein of 41 kDa b	Q9SA52	Chloroplast stem-loop binding protein of 41 kDa b (CSP41-b)	85.8		stroma
**A0A1S3ZRR1**	**Nucleoid-associated protein At4g30620**	**Q9M098**	**Nucleoid-associated protein At4g30620**	**76.4**	**1**	**stroma**
**A0A1S4CGA5**	**Pentatricopeptide repeat-containing protein At4g30825**	**O65567**	**Pentatricopeptide repeat-containing protein At4g30825**	**63.8**	**9**	**stroma**
A0A1S3YRF9	Ribosome-recycling factor	Q9M1X0	Ribosome-recycling factor (RRF)	64.4	0	stroma
*Amino acid biosynthesis*						
A0A1S4CUE0	Ferredoxin-dependent glutamate synthase	Q9ZNZ7	Ferredoxin-dependent glutamate synthase 1 (Fd-GOGAT 1)	83.9		stroma
A0A1S3YTZ2	Ketol-acid reductoisomerase	Q05758	Ketol-acid reductoisomerase	84.8		stroma
A0A1S4APF3	Ornithine carbamoyltransferase	O50039	Ornithine carbamoyltransferase	75.3		stroma
*Response to stress*						
A0A1S4CDL2	Protein CutA	P93009	Protein CutA	69.6	1	inter-membrane
A0A1S4A194	Soluble inorganic pyrophosphatase 6	Q9LXC9	Soluble inorganic pyrophosphatase 6 (Ppase 6)	74.6	0	stroma
Chlorophyll synthesis						
**A0A1S4C5X4**	**Oxygen-dependent coproporphyrinogen-III oxidase**	**Q9LR75**	**Coproporphyrinogen-III oxidase 1 (CPOX)**	**80.4**	**2**	**stroma**
*Photorespiration*						
**A0A1S3X073**	**Phosphoglycolate phosphatase 1B**	**P0DKC4**	**Phosphoglycolate phosphatase 1B**	**66.8**	**4**	**stroma**
*PSII assembly*						
A0A1S4DN09	Photosystem II repair protein PSB27-H1	Q9LR64	Photosystem II repair protein PSB27-H1	64.5	0	thylakoid ls
A0A1S4DKC9	Photosystem II stability/assembly factor HCF136	O82660	Photosystem II stability/assembly factor HCF136	78.3	0	thylakoid ls
*Sulfur metabolism*						
A0A1S4CCJ9	Cysteine synthase	P47999	Cysteine synthase	83.5		stroma
*Unknown function*						
A0A1S4BU42	Thylakoid lumenal protein TL20.3	Q8H1Q1	Thylakoid lumenal protein TL20.3	75.8		lumen

## Data Availability

Data is contained within the article and the Supplementary Material.

## Supplementary Materials

The following supporting information can be downloaded at: https://www.mdpi.com/article/10.3390/antiox11101979/s1, Table S1. List of primers used for monocysteinic Trxs expression; Table S2. Proteomic analysis of Trx f candidate targets; Table S3. Proteomic analysis of Trx m candidate targets; Table S4. Proteomic analysis of NTRC candidate targets; Table S5. Amino acid sequences of new potential identified targets of Trx f, Trx m and NTRC; Figure S1. Silver staining and western blot analysis of fractions collected after Trx purification.
