# Supplementary material for: New In Vivo Approach to Broaden the Thioredoxin Family Interactome in Chloroplasts
Source: Antioxidants (Basel). 2022 Oct 4;11(10):1979. doi: 10.3390/antiox11101979 (PMC9598788; doi:10.3390/antiox11101979)
Supplement: Supplementary file 1 [file antioxidants-11-01979-s001.zip › Supplementary material.pdf]

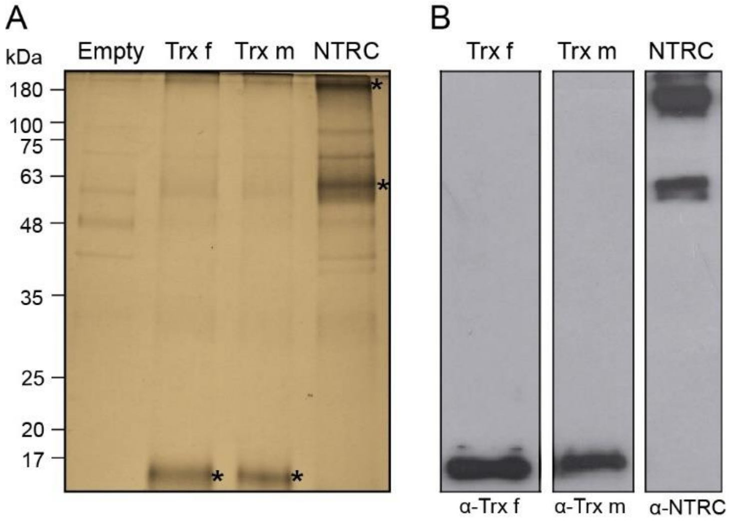

Figure S1. Silver staining and western blot analysis of fractions collected after Trx purification. *N. benthamiana* plants agroinfiltrated with the monocysteinic mutant variants of Trx f, Trx m and NTRC or the empty vector as control were eluted with imidazole and subjected to SDS-PAGE. Similar volumes were separated in 13.5% acrylamide gels and then proteins were visualized by silver staining (A) or immunoblotted (B) with specific anti-Trxf, anti-Trx m (1: 5000) or anti-NTRC (1:1000) antibodies. Asterisks (*) indicate the corresponding overexpressed Trx for each fraction.

Table S1. List of primers used for monocysteinic Trxs expression.

	Site-directed mutagenesis (5'-3') [a]
	C47S-f: CTCAGTGGTGTGGTCCTAGCAAAGTG
	C47S-r: CACTTTGCTAGGACCACACCACTGAG
	Full gene length (5'-3') [b,c]
Trxf-C47S	Trxf-f: ATGCAGGTGTGGAGCTCCGATGCTACTGCTAC
	Trxf-r: tctagaTTA*ATGGTGATGGTGATGGTG*ACTTGACCGCACATCCTC
	Rubisco small subunit transit peptide (5'-3') [b]
	Rbcs-f: ccatggCTTCCTCAGTTCTTTCC
	Rbcsf-r: ATCGGAGCTCCACACCTGCATGCATTGCAC
	Site-directed mutagenesis (5'-3') [a]
	C40S-f: CTCCGTGGTGTGGTCCAAGCCGAATG
	C40S-r: CATTCGGCTTGGACCACACCACGGAG
	Full gene length (5'-3') [b,c]
Trxm-C40S	Trxm-f: ATGCAGGTGTGGGAAGCGCAAAATACTGCC
	Trxm-r: tctagaTTA*ATGGTGATGGTGATGGTG*CAAGAATTTCTCTATGCAGG
	Rubisco small subunit transit peptide (5'-3') [b]
	Rbcs-f: ccatggCTTCCTCAGTTCTTTCC
	Rbcsm-r: TTGCGCTTCCCACACCTGCATGCATTGCAC
	Site-directed mutagenesis (5'-3') [a]
	C457S-f: CACCAACATGTGGCCCCAGTAGGACTC
	C457S-r: AGTCCTACTGGGGCCACATGTTGGTG
	Full gene length (5'-3') [b,c]
NTRC-C457S	NTRC-f: ATGCAGGTGTGGTCTTCTTCAGGAGGCGAG
	NTRC-r: tctagaTTA*ATGGTGATGGTGATGGTG*TTTATTGGCCTCAATGAATTC
	Rubisco small subunit transit peptide (5'-3') [b]
	Rbcs-f: ccatggCTTCCTCAGTTCTTTCC
	RbcsNTRC-r: TGAAGAAGACCACACCTGCATGCATTGCAC

[a] Single nucleotide changes to replace Cys with Ser are underlined

[b] Restriction sites are indicated in lowercase

[c] Italics indicate 6xHis tag sequence

Table S2. Proteomic analysis of Trx f candidate targets. Specified criteria: at least two unique peptides, p-value lower than 0.05, and a Log2 fold change >1.8.

Accession	Description	Subcellular localization	Unique peptides	Peptide count	Confidence score	p-value	Fold Change
A0A1S3Z4R9	12-oxophytodienoate reductase 3-like	Peroxisome	2	2	12	0.00	2.42
L7UU40	26S protease regulatory subunit 8 homolog A-like	Nucleus, cytoplasm	2	2	13	0.00	3.33
A0A1S4BDA7	2-Cys peroxiredoxin BAS1	Chloroplast	9	18	419	0.00	7.79
A0A1S4DBQ8	40S ribosomalprotein S15a-1	Cytoplasm	3	3	37	0.05	2.49
A0A1S4DHJ8	5'-adenylylsulfate reductase 2	Chloroplast	4	4	67	0.00	5.80
Q6T7F3	Amidophosphoribosyl transferase	Chloroplast	9	9	150	0.01	2.13
A0A1S3ZQZ9	Annexin	Unknown	2	5	46	0.00	2.74
A0A1S4APY2	Aspartate--tRNA ligase 2	Cytoplasm	3	3	35	0.00	2.10
A0A1S4A4L1	Aspartyl protease family protein 1-like	Plasma membrane	2	2	27	0.00	1.83
A0A1S4D2D2	BTB/POZ domain-containing protein At1g03010-like isoform X2	Unknown	2	2	16	0.00	1.96
A0A1S4B8Q6	Cis-abienol synthase	Chloroplast	2	2	13	0.01	2.26
A0A1S3YJX2	Dihydropyrimidinase isoform X1	Endoplasmic reticulum	2	2	15	0.00	14.95
A0A1S3XYZ9	GDSL esterase/lipase 2-like	Secreted	2	7	82	0.00	4.99
A0A1S4ATB8	Glyceraldehyde-3-phosphate dehydrogenase	Chloroplast	8	20	294	0.00	3.19
A0A1S4AKJ1	Golgin candidate 1-like	Golgi apparatus	2	2	17	0.01	1.98
A0A1S3Y7P2	Patatin-like protein 3	Plasma membrane	3	3	23	0.00	1.90
A0A1S4C620	Peptide methionine sulfoxide reductase-like	Cytosol	3	3	44	0.00	4.84
A0A1S4DGW 7	Peptidyl prolyl isomerase	Chloroplast	3	3	28	0.01	1.86
A0A1S4B900	Peroxiredoxin Q	Chloroplast	11	14	264	0.00	2.92
A0A1S4DK72	Peroxiredoxin-2B-like	Cytoplasm	3	8	166	0.00	4.47
A0A1S4D678	Peroxiredoxin-2E-2	Chloroplast	6	8	155	0.00	2.39
A0A1S4CIH3	PGR5-like protein 1A	Chloroplast	2	2	27	0.00	1.81
A0A1S4CZ71	Phosphoglucan waterd ikinase	Chloroplast	2	2	23	0.00	3.69
A0A1S3YLT9	Probable endo-1,3(4)-beta-glucanase ARB_01444 isoform X3	Unknown	2	3	44	0.00	11.18
A0A1S4D5H7	Proline-rich receptor-like protein kinase PERK3	Plasma membrane	2	2	12	0.01	2.35
A0A1S4A0A5	Protein FLX-like 3 isoform X1	Unknown	2	2	10	0.00	2.64
A0A1S4C9Y8	Putative late blight resistance protein homolog R1A-3	Unknown	2	2	16	0.00	2.46
A0A1S4BBZ3	Putative uridine kinase C227.14 isoform X2	Cytosol	2	2	24	0.00	3.48
A0A140G1V5	Ribosomal protein S3	Chloroplast	2	2	13	0.01	2.53
A0A1S3ZVC8	Obg-like ATPase 1	Cytosol	7	7	104	0.00	2.77
A0A1S4A3L9	Ribulose bisphosphate carboxylase/oxygenase activase 1	Chloroplast	2	15	248	0.01	2.13
A0A1S4BTX2	Stress response protein NST1-like	Unknown	2	2	19	0.01	1.94
A0A1S4D2Y9	Thioredoxin-like 4	Chloroplast	7	7	94	0.00	5.02
A0A1S4CQQ3	Thylakoid lumenal 29 kDa protein	Chloroplast	2	4	55	0.00	1.90
A0A1S4AFW1	Tryptophan--tRNA ligase, cytoplasmic-like isoform X2	Cytoplasm	2	2	28	0.00	2.58
A0A1S4DKS1	UBP1-associated protein 2B-like	Nucleus	5	6	68	0.02	2.97
A0A1S4B454	Uncharacterized protein LOC107804229	Unknown	2	2	25	0.00	5.14
A0A1S4CK21	Uncharacterized protein LOC107819880	Unknown	2	2	14	0.00	8.33
A0A1S4CR05	Uridine kinase	Chloroplast	2	2	18	0.00	2.50

Table S3. Proteomic analysis of Trx m candidate targets. Specified criteria: at least two unique peptides, p-value lower than 0.05, and a Log2 fold change >1.8.

Accession	Description	Subcellular localization	Unique peptides	Peptide count	Confidence score	p-value	Fold Change
A0A1S3Z4R9	12-oxophytodienoate reductase 3-like	Peroxisome	2	2	12	0.00	2.01
L7UU40	26S protease regulatory subunit 8 homolog A-like	Nucleus	2	2	13	0.00	2.80
A0A1S4A3V7	2-Cys peroxiredoxin BAS1	Chloroplast	7	17	350	0.05	3.75
A0A1S4DBQ8	40S ribosomalprotein S15a-1	Cytoplasm	3	3	37	0.00	1.95
A0A1S3ZK73	40S ribosomal protein S25-like	Cytosol	2	3	21	0.00	1.93
A0A1S4DL03	50S ribosomal protein L18	Chloroplast	2	2	27	0.01	3.23
A0A1S4DHJ8	5'-adenylylsulfate reductase 2	Chloroplast	4	4	67	0.00	19.89
A0A1S3XPC1	Altered inheritance of mitochondria protein 32-like	Unknown	2	2	17	0.01	1.85
Q6T7F3	Amidophosphoribosyl transferase	Chloroplast	9	9	150	0.00	2.94
A0A1S3ZQZ9	Annexin	Unknown	2	5	46	0.04	1.88
A0A1S4B8Q6	Cis-abienol synthase	Chloroplast	2	2	13	0.00	2.14
A0A1S3YJX2	Dihydropyrimidinase isoform X1	Endoplasmic reticulum	2	2	15	0.00	4.52
A0A1S3XYZ9	GDSL esterase/lipase 2-like	Secreted	2	7	82	0.01	4.66
A0A1S4A969	Glutathione peroxidase	Chloroplast	6	6	69	0.02	2.66
A0A1S4AKJ1	Golgin candidate 1-like	Golgi apparatus	2	2	17	0.01	1.99
A0A1S4DEY3	Granule-bound starch synthase 1	Chloroplast	2	3	22	0.00	2.71
A0A1S4B4B2	Histone-lysine N-methyl transferase setd3-like	Unknown	3	3	46	0.00	3.68
A0A1S3YQS9	Malate dehydrogenase (NADP)	Chloroplast	12	12	171	0.00	1.83
A0A1S4BQQ0	Nudix hydrolase 8-like	Unknown	2	2	24	0.00	9.46
A0A1S4B8P1	Peptide methionine sulfoxide reductase-like	Chloroplast	7	7	125	0.00	6.11
A0A1S4C620	Peptide methionine sulfoxide reductase-like	Cytosol	3	3	44	0.00	16.71
A0A1S4B900	Peroxiredoxin Q	Chloroplast	11	14	264	0.01	4.66
A0A1S4DK72	Peroxiredoxin-2B-like	Cytoplasm	3	8	166	0.02	2.87
A0A1S4CIH3	PGR5-like protein 1A	Chloroplast	2	2	27	0.03	5.09
A0A1S4CZ71	Phosphoglucan waterdikinase	Chloroplast	2	2	23	0.00	2.41
A0A1S4D5H7	Proline-rich receptor-like protein kinase PERK3	Plasma membrane	2	2	12	0.00	2.10
A0A1S4A0A5	Protein FLX-like 3 isoform X1	Unknown	2	2	10	0.01	2.10
A0A1S4C9Y8	Putative late blight resistance protein homolog R1A-3	Unknown	2	2	16	0.01	2.23
A0A1S4BBZ3	Putative uridine kinase C227.14 isoform X2	Cytosol	2	2	24	0.01	3.53
A0A140G1V5	Ribosomal protein S3	Chloroplast	2	2	13	0.00	2.44
A0A1S4A3L9	Ribulose bisphosphate carboxylase/oxygenase activase 1	Chloroplast	2	15	248	0.02	2.16
A0A1S3YEX0	Sedoheptulose-1,7-bisphosphatase	Chloroplast	6	16	246	0.01	2.11
A0A1S4ASD9	Thioredoxin-like 2	Chloroplast	2	3	49	0.01	2.65
A0A1S4D2Y9	Thioredoxin-like 4	Chloroplast	7	7	94	0.00	8.60
A0A1S4AFW1	Tryptophan--tRNA ligase, cytoplasmic-like isoform X2	Cytoplasm	2	2	28	0.00	1.95
A0A1S4DKS1	UBP1-associated protein 2B-like	Nucleus	5	6	68	0.00	2.22
A0A1S3Y1A3	Uncharacterized protein LOC107771070	Unknown	2	2	15	0.00	5.43
A0A1S3ZX69	Uncharacterized protein LOC107791394	Unknown	4	4	68	0.01	3.63
A0A1S4CK21	Uncharacterized protein LOC107819880	Unknown	2	2	14	0.02	2.93
A0A1S3Y9V3	UPF0061 protein azo1574-like	Unknown	2	3	29	0.00	3.20
A0A1S4CR05	Uridine kinase-like protein 1	Chloroplast	2	2	18	0.01	1.96

Table S4. Proteomic analysis of NTRC candidate targets. Specified criteria: at least two unique peptides, p-value lower than 0.05, and a Log2 fold change >1.8.

Accession	Description	Subcellular localization	Unique peptides	Peptide count	Confidence score	p-value	Fold Change
A0A1S4AH01	10 kDa chaperonin-like	Chloroplast	4	4	68	0.00	3.24
Q43590	1-amniocyclopropane-1-carboxylate oxidase	Unknown	8	8	145	0.00	2.19
A0A1S3XWD3	2,3-bisphosphoglycerate-independent phosphoglycerate mutase	Cytoplasm	6	6	102	0.00	2.38
A0A077DBL2	20 kDa chaperonin	Chloroplast	4	4	75	0.00	4.59
A0A1S4CYJ5	29 kDa ribonucleoprotein A	Chloroplast	6	6	144	0.00	2.24
A0A1S4A3V7	2-Cys peroxiredoxin BAS1	Chloroplast	3	15	256	0.00	13.15
A0A1S3XX03	31 kDa ribonucleoprotein	Chloroplast	3	3	47	0.01	4.42
A0A1S3ZYM9	5-methyltetrahydropteroyl triglutamate homocysteine methyltransferase-like	Cytosol	4	12	168	0.01	2.51
A0A1S4DLA0	60S ribosomal protein L35-like	Cytosol	2	2	34	0.00	2.79
P17514	Acidic endochitinase Q	Vacuole	7	7	150	0.00	5.53
Q9AT16	Ankyrin-repeat protein HBP1	Nucleus	5	5	82	0.00	5.53
A0A140G1P8	ATP synthase CF0 B subunit	Chloroplast	9	10	113	0.00	2.47
A0A1S4CSA5	ATP synthase delta chain	Chloroplast	3	3	41	0.00	3.71
P00823	ATP synthase subunit alpha	Chloroplast	2	35	642	0.01	2.23
A0A140G1S2	ATP synthase subunit beta	Chloroplast	27	31	689	0.00	3.42
A0A1S4C3F4	Calreticulin isoform X2	Endoplasmic reticulum	5	5	89	0.01	2.87
A0A1S4B1Q8	CBS domain-containing protein CBSX1	Chloroplast	2	2	20	0.00	3.05
A0A1S4DIE1	Chlorophyll a-b binding protein 13	Chloroplast	5	5	100	0.00	7.68
A0A1S4BMB0	Chlorophyll a-b binding protein 36	Chloroplast	4	8	124	0.00	5.04
A0A1S4CBW5	Chlorophyll a-b binding protein 8	Chloroplast	7	7	163	0.04	2.13
Q40512	Chlorophyll a-b binding protein	Chloroplast	3	3	55	0.00	3.89
Q0PWS7	Chlorophyll a-b binding protein	Chloroplast	6	6	101	0.00	4.76
Q0PWS6	Chlorophyll a-b binding protein	Chloroplast	4	4	106	0.00	6.29
A0A1S3Z334	Chloroplast stem-loop binding protein of 41 kDa b	Chloroplast	32	32	611	0.00	2.10
Q9LRI2	Cysteine protease	Vacuole	4	4	55	0.01	6.00
A0A1S4CCJ9	Cysteine synthase	Mitochondrion	7	10	213	0.01	2.62
A0A140G1T3	Cytochrome b559 subunit alpha	Chloroplast	2	2	22	0.00	3.87
A0A1S4ACF7	Cytochrome b561 and DOMON domain-containing protein At3g25290-like	Unknown	2	2	18	0.00	3.03
A0A1S3XVT6	Cytochrome b_6	Chloroplast	2	2	38	0.01	2.86
A0A1S4B832	Cytochrome b_6f complex iron-sulfur subunit	Chloroplast	5	5	84	0.00	2.14
A0A1S4CNA9	Cytochrome c-like	Mitochondrion	2	2	21	0.00	3.10
A0A140G1S8	Cytochrome f	Chloroplast	12	12	241	0.01	3.03
A0A1S4BMJ4	Earlynodulin-like protein 2	Plasma membrane	2	4	53	0.00	3.95
A0A1S4D7J1	Endochitinase B	Vacuole	2	2	49	0.01	5.57
A0A1S4CDZ9	Fasciclin-like arabinogalactan protein 1	Plasma membrane	2	2	61	0.00	2.30
A0A1S4AMD8	Fasciclin-like arabinogalactan protein 8	Plasma membrane	2	2	33	0.00	4.52
A0A1S3YVN4	Ferredoxin	Chloroplast	5	5	148	0.01	4.73
A0A1S4CUE0	Ferredoxin-dependent glutamate synthase	Chloroplast	2	4	75	0.01	2.34
A0A1S4B5N2	Ferredoxin-thioredoxin reductase	Chloroplast	4	4	40	0.00	2.39
A0A1S4A023	Fructose-1,6-bisphosphatase	Chloroplast	6	6	102	0.00	1.90
A0A1S3YQI2	Fructose-bisphosphate aldolase	Cytosol	7	8	156	0.00	2.21
P23547	Glucan endo-1,3-beta-glucosidase, acidic isoform GI9	Secreted	3	3	41	0.00	2.31
A7XAQ5	Glucose-1-phosphate adenylyltransferase	Chloroplast	4	4	72	0.00	1.84
A0A1S4CUX2	Glutaredoxin	Cytoplasm	3	4	55	0.00	2.38
A0A1S4A969	Glutathione peroxidase	Chloroplast	4	4	43	0.00	4.06
A0A1S3Y8V8	Glutathione S-transferase DHAR3	Chloroplast	4	4	35	0.00	2.86
A0A1S3XJF2	Glyceratede hydrogenase	Peroxisome	5	5	84	0.00	3.30
A0A1S3YN86	Glycerophosphodiester phosphodiesterase GDPDL3-like	Plasma membrane	2	2	31	0.00	3.21
A0A1S3YRT4	Glycine cleavage system H protein	Mitochondrion	5	5	106	0.00	2.23
O82077	Glycolate oxidase	Peroxisome	3	7	149	0.03	2.62
A0A1S4BVY0	GrpE protein homolog	Mitochondrion	6	6	74	0.00	1.91

A0A1S3ZX72	Haloacid dehalogenase-like hydrolase domain-containing protein At3g48420	Chloroplast	5	5	49	0.01	1.94
A0A1S3YTZ2	Ketol-acid reductoisomerase	Chloroplast	2	2	20	0.01	2.47
A0A1S3YXG6	Malate dehydrogenase	Mitochondrion	9	17	376	0.00	5.91
A0A1S3Y6M1	Malate dehydrogenase	Cytoplasm	2	7	149	0.00	3.09
A0A1S3ZW53	Malate dehydrogenase	Mitochondrion	4	12	251	0.00	7.67
A0A1S3YUT4	Monodehydroascorbate reductase	Peroxisome	6	6	94	0.01	2.62
A0A1S4BWK5	NADP-dependent glyceraldehyde-3-phosphate dehydrogenase-like	Cytoplasm	4	4	82	0.00	2.19
A0A1S4CLH1	Nascent polypeptide-associated complex subunit alpha-like protein 1	Cytosol	2	4	53	0.00	4.69
A0A1S3ZRR1	Nucleoid-associated protein At4g30620	Chloroplast	2	2	30	0.00	5.47
A0A1S4APF3	Ornithine carbamoyl transferase	Chloroplast	2	2	40	0.00	3.37
Q84QE8	Oxygen evolving complex 33 kDa photosystem II protein	Chloroplast	10	18	316	0.00	3.87
A0A1S4C5X4	Oxygen-dependent coproporphyrinogen-III oxidase	Chloroplast	3	3	34	0.01	1.96
A0A1S3ZHB8	Oxygen-evolving enhancer protein 1	Chloroplast	6	21	431	0.00	4.60
A0A1S4BMY9	Oxygen-evolving enhancer protein 2-2	Chloroplast	8	9	215	0.00	3.86
A0A1S3XRM3	Oxygen-evolving enhancer protein 3-2	Chloroplast	16	17	266	0.00	3.89
A0A1S4CGA5	Pentatricopeptide repeat-containing protein At4g30825	Chloroplast	2	2	8	0.00	73.37
A0A1S4AWT3	Peptidyl-prolyl cis-trans isomerase	Chloroplast	7	8	124	0.00	4.14
A0A1S3ZH83	Peptidyl-prolyl cis-trans isomerase CYP38	Chloroplast	4	4	45	0.00	2.07
A0A1S3XJV2	Peptidyl-prolyl isomerase	Chloroplast	6	6	76	0.00	3.63
A0A1S4A107	Peroxidase	Secreted	2	2	33	0.00	7.12
A0A1S4CAV2	Peroxidase	Secreted	4	4	58	0.00	6.06
A0A1S3XDP9	Peroxiredoxin-2B-like	Cytoplasm	2	5	82	0.01	2.06
A0A1S4ANB9	Peroxisomal (S)-2-hydroxy-acid oxidase-like	Peroxisome	3	7	140	0.00	2.91
A0A1S3Z828	Phosphoglycerate kinase	Cytoplasm	5	12	194	0.00	3.50
A0A1S3X073	Phosphoglycolate phosphatase 1B	Chloroplast	2	3	27	0.01	3.86
Q1W375	Phosphomannomutase	Cytoplasm	2	2	18	0.01	2.09
A0A140G1X0	Photosystem I iron-sulfur center	Chloroplast	10	10	209	0.00	4.33
A0A140G1R3	Photosystem I P700 chlorophyll a apoprotein A1	Chloroplast	7	7	70	0.00	6.14
A0A140G1R2	Photosystem I P700 chlorophyll a apoprotein A2	Chloroplast	4	4	52	0.01	5.42
A0A1S3ZIE1	Photosystem I reaction center subunit II	Chloroplast	20	20	400	0.00	3.67
A0A1S4CFV4	Photosystem I reaction center subunit IV A	Chloroplast	4	4	75	0.00	4.29
A0A1S4CYN6	Photosystem I reaction center subunit IV B	Chloroplast	6	6	113	0.00	4.46
D2K7Z2	Photosystem I reaction center subunit	Chloroplast	5	6	118	0.00	2.26
A0A1S4CR54	Photosystem I reaction center subunit VI-1	Chloroplast	3	3	40	0.03	2.10
A0A1S4BQS3	Photosystem I reaction center subunit XI	Chloroplast	2	2	59	0.01	4.77
A0A1S3YQ87	Photosystem II 22 kDaprotein	Chloroplast	2	2	44	0.04	2.39
A0A140G1Q8	Photosystem II CP43 reaction center protein	Chloroplast	14	16	294	0.00	4.94
A0A140G1U3	Photosystem II CP47 reaction center protein	Chloroplast	26	26	502	0.00	4.96
A0A140G1Q7	Photosystem II D2 protein	Chloroplast	7	7	186	0.00	6.16
A0A140G1P2	Photosystem II protein D1	Chloroplast	2	2	36	0.01	4.37
A0A1S4DN09	Photosystem II repair protein PSB27-H1	Chloroplast	6	6	129	0.00	4.49
A0A1S4DKC9	Photosystem II stability/assembly factor HCF136	Chloroplast	7	7	114	0.01	3.49
A0A1S4A1K3	Plastocyanin	Chloroplast	5	5	78	0.00	4.39
A0A1S3ZZS2	Probable L-ascorbate peroxidase 6, chloroplastic isoform X2	Chloroplast	3	3	70	0.00	2.34
A0A1S4B0C4	Probable protein Pop3 OS	Cytosol	2	2	17	0.00	2.32
A0A1S3Z1X1	Probable ribose-5-phosphate isomerase 3	Chloroplast	6	6	121	0.01	2.63
A0A1S4CDL2	Protein CutA	Chloroplast	2	2	22	0.03	2.16
A0A1S3YRF9	Ribosome-recycling factor	Chloroplast	5	5	82	0.00	4.29
A0A140G1S3	Ribulose bisphosphate carboxylase largechain	Chloroplast	50	52	1126	0.00	2.11
A0A1S4DIY1	Rubisco large subunit-binding protein subunit beta	Chloroplast	3	7	101	0.00	1.90
A0A1S4ABS2	Serine—glyoxylate aminotransferase	Peroxisome	7	7	102	0.00	4.36
A0A1S4A194	Soluble inorganic pyrophosphatase 6	Chloroplast	3	3	36	0.00	3.97
A0A1S3ZTX1	Superoxide dismutase [Cu-Zn]	Nucleus, cytosol	3	3	53	0.00	3.47
W0KRH1	Superoxide dismutase	Chloroplast	3	3	44	0.00	2.39
A0A1S4CCB3	Thioredoxin-like	Chloroplast	2	6	82	0.00	2.28
A0A1S4BU42	Thylakoid luminal protein TL20.3	Chloroplast	3	3	72	0.00	3.78
A0A1S3X2Z0	Triosephosphate isomerase	Chloroplast	3	4	44	0.02	3.25
A0A1S3XK44	Uncharacterized protein At4g15545-like	Unknown	2	2	17	0.01	2.06

A0A1S3Z0H0	Uncharacterized protein LOC107781661	Nucleus	2	2	24	0.00	3.24
A0A1S4BZK6	Uncharacterized protein LOC107813570	Mitochondrion	4	4	58	0.00	3.03
A0A1S4D357	Uncharacterized protein LOC107825453	Nucleus	2	7	107	0.01	2.12
A0A1S4D8Y5	Uncharacterized protein LOC107827263	Unknown	4	5	59	0.00	5.22
A0A1S4DQW8	Uncharacterized protein LOC107832223	Chloroplast	2	2	31	0.02	2.13
A9CM22	Voltage-dependent anion channel	Mitochondrion	4	5	54	0.00	3.08
A0A097BTV9	V-type proton ATPase catalytic subunit A	Vacuole	2	52	1059	0.00	2.06
A0A1S4AP74	V-type proton ATPase subunit a	Vacuole	8	8	119	0.02	2.88
A0A1S3ZYF9	V-type proton ATPase subunit	Vacuole	3	3	34	0.00	2.91
A0A1S4DC16	V-type proton ATPase subunit H	Vacuole	2	2	19	0.00	2.12
A0A1S4AQ72	Xyloglucanendotransglucosylase/hydrolase	Secreted	4	4	59	0.00	2.00

Table S5. Amino acid sequences of new potential identified targets of Trx f, Trx m and NTRC. Conserved cysteines (C) are indicated in bold and underlined.

Trx f and Trx m targets

>A0A1S4D2Y9_Thioredoxin-like 4
MQRLGILDWQHNLSFRSINLLDGEPAHTLLSSLSFRSSSGSSHSKTRLCSNLRHADLNVTLQSLVRQCSEIQNAVHDNIEELLDEEDDLCPVECVREFKT
DEEFSIILEKAKKAGSLVVVDFYRTA**C**GS**C**KYIEQGFAKLCRGAGDEQAPVIFLKHNVIDEYDEQSEVAERLRIKTVPLFHFYKNGVLLEAFPTRDKERILA
AILKYSAPASADV

>A0A1S4CZ71_Phosphoglucan water dikinase
MDSLHFSHCYSTLNAKKRQPQNQISKQFTVLPQLSQTSVKNSILLPRKKLGFLMDYKGRGTGIVRAVSSVETREKHQKGKNNKNTEKVQLRVRLKHQV
EFGEHIAVLGSAKELGSWKKNIMMDWTENGWISELELPAGESLEYKFVIVGKDKNMLWENGSNRILRLPEGGSFELV**C**QWNVTDEPVNLLQLDPFVVE
EEVEAASDNGATITGEAAVLDAVTSPFVEQWQGRAASFVRSKDELDSEKNRKWDTSGLTGISLKLVEGDKNARNWWRKLEVVRELVVENMDSSQRLE
ALTYAAVYLKWINTGQIP**C**LEDGGHHRPNRHAEISRLIFREVEKVLSRRDTTLQEILVIRKMQP**C**LPSFKAEFTQSVPLTRIRDIAHRNDIPHDLKQEIKHTI
QNKLHRNAGPEDLVSTEAMLERITKKPGQYSEAFVEQFKIFHNELKDFFNAGSLDEQLESIRESLDESKSSMLSSFLESKKGLAILDEKHNVSETERMGS
LVRTINSLNALREVIVKGLESGLRNDAPDAAIAMRQKWRL**C**EIGLEDYAFVLLSRFVNAVEARGGADSLAENVAQKNVSSWNDPIGALNVGIQQLGLSG
WKPEE**C**KAVGNELLSWKKRGLSETEGSEDGKTIWALRLKATLDRSRRLTEEYSETLLQIFPEKVQILGKSLAIPENSVRTFTEAEIRAGVVFQVSKLATLL
LKATRRTIGSSGWDVLVPGDAFGQLIQVDRIVPGTLPSSATGPVILVVNKADGDEEVTAAGSNISGVVLLQELPHLSHLGVRARQEKVVFVT**C**DDDDKV
SDIRELLGKYVRLEASSTGVKLTSSSSEKGTGVSQKKHLSVTASSTSTASSDSSASSIAVKSSHSKEVNNFS**C**R

>Q6T7F3_Amidophosphoribosyltransferase
MAATVSTASAAATNKSPLSQPLDKPFCSLSQKLLSLSPKTHPKPYRTLITASSKNPLNDVISFKKSADNTLDSYFDDDDKPREE**C**GVVGIYGDSEASRL**C**
YLALHALQHRGQEGAGIVAVNDDVLKSITGVGLVSDVFNESKLDQLPGDMAIGHVRYSTAGSSMLKNVQPFVASYKFGSVGVAHNGNLVNYKLLRSEL
EENGSIFNTSSDTEVVLHLIAISKARPFLLRIVEA**C**EKIEGAYSMVFVTEDKLVAVRDPHGFRPLVMGRRSNGAVVFASET**C**ALDLIEATYEREVNPGEVV
VVDKDGVQSI**C**LMPHPERKS**C**IFEHIYFALPNSVVFGRSVYESRRAFGEILATEAPVECDVVIAVPDSGVVAALGYAAKAGVPFQQGLIRSHYVGRTFIEP
SQKIRDFGVKLKLSPVRAVLEGKRVVVVDDSIVRGTTSSKIVRLLKEAGAKEVHMRIASPPIIAS**C**YYGVDTPSSDELISNRMSVEEIKEFIGSDSLAFLPM
DSLNKLLGNDSKSF**C**YA**C**FSGNYPVEPTGKVKRIGDFMDDGLSGDMDSIDGGWLPGSSRVQKTILNEVRTS

>A0A140G1V5_Ribosomal protein S3
MGQKINPLGFRLGTTQGHHSLWFSQPKNYSEGLQEDQKIRD**C**IKNYVQKNMRTSSGVEGIARIEIQKRIDLIQVIIFMGFPKLLIESRPRGIEELQTTLQKE
FH**C**VNRKLNIAVTRIAKPYGNPNILAEFIAGQLKNRVSFRKAMKKAIELTEQADTKGIQIQIAGRIDGKEIARVEWIREGRVPLQTIRAKIDY**C**SYTVRTIYGV
LGIKIWIFLDEE

>A0A1S4CR05_Uridine kinase
MAPVPEETTAIDYVMEAASGAHFSGLRFDGLLTSGSASPRASPTHTPTHFSTTIPLDSTTPKQPFVIGVSGGTASGKTTVCDMIIQQLHDHRVVLVNQDS
FYRGLTPEEMKRVHEYNFDHPDAFDTEQLLECVEKLKSGLSVQVPIYDFKTHQRCSDSFRQVNASDVIILEGILVFHDSRVRNLMNMKIFVDTDADVRLA
RRIRRDTVERGRDINSVLEQYAKFVKPAFDDFVLPSKKYADVIIPRGGDNHVAIDLITQHIRTKLGQHDLCKIYPNVYVIQSTFQIRGMHTLIRDKDISKHDF
VFYSDRLIRLVVEHGLGHLPFTEKQIVTPTGSVYTGVDFCKKLCGVSIVRSGESMENALRACCKGIKIGKILIHRDGDNGKQLIYEKLPKDISERHVLLLDP
VLATGNSANQAIELLIQKGVPESHIIFLNLISAPEGIHCVCKRFPSLKIVTSEIDLALNEEYRVIPGLGEFGDRYFGTDD

>A0A1S4B8Q6_Cis-abienol synthase
MILGYGSIIILPFSHHKLGNGKLCSSTENTICQRPCRGVRCSYSIASSLDGFDEAKERIKKSFQKVELSPSSYDTAWVAMIPSINSVNQPCFPQCLDWILE
NQREDGSWGLNPSHSLLVKDSLSSTLACLLALRKWGVGDNQVQGGLVFIEKHGWAVDNKDLISPVGFEIIFPSMIKYAEKMNLNLPLDPDIVNLAIRNRD
LAIERALQNDFKGNIANFEYMAEGLSELCQWKEIMVHQRDNGSLFDSPATTAAALIYHQHDEKCFEYLNTILKLHKNWVPTIYPTKIHSLLCLVDTLQSLG
VDRHFKTEIENVLDEIYRLWQQKNEEIFSNVAHCALAFRLLRMSNYEVSPEELVEFVDEVHFFSTSGKFTSHFEILELHKASQLAIHGKDHILDKISNWTG
SFMEQKLLTYDYIDRMSKNEAEFALRKFYATYGRVENRRYNEAYEVNNFKILKAAYRSPTINSIDLLRFSKQDFNLCQAQHQEELQQLKRWDGNYSTVQ
FHSERIKIFFSALYKTIEELAAKANIKQGQCIKEHFINLWLDLLKNMLVEFEWWRNQTTPSIEEYLSVACETIGVRCITLITQCLLGPKLSNDVLQSSEMSAL
CNCTSMVARLLNDVGSYKREEAESSPTNIVSILINQSEGKISEEEAIKHAKEMLENKRRELLGMVLIQRKGSQLPQVCKDIFWKTCKSSYFAYSDGDEFR
FPEEILKNRINELLFKPLKS

Trx m targets

>A0A1S4ASD9_Thioredoxin-like 2
MADIMGFSLNSLRFSSSSASNSSLLTSFSSSLNSIQTSNQKLHKRAVSLSDSPSTSVDFVSGITLRPNKRFPAVKVHATVTRTEEPKWWERNAGPNMVD
IHSTKEFLDALSQAGERLVIVEFYGTW**C**AS**C**KALFPKLCRIAQENPEIMFLKVNFDANKPMCKALNVKVLPYFHFYRGADGQLESFSCSLAKFQKIKDAIQ
LHNTARCSIGPPIGVGDLTLELLSGTK

>A0A1S4DEY3_Starch synthase
MASITASHFVSRSSNVCSGAASVDTRANLSQIGLRNHALTHNGLRAVNKVDMLQSRTNTKVTAKKSSKQVSGTEMERPSGTIVCGKGMNVILVGTEVG
PWSKTGGLGDVLGGLPPALAARGHRVMTISPRYDQYKDAWDTSVVVEIKVGDKIEIVRFFH**C**YKRGVDRVFVDHPMFLEKVWGKTAAKIYGPKAGQD
YLDNELRFSLL**C**QAALEAPRVLNLNCSEYFSGPYGEDVVFIANDWHTALLPCYLKSMYQSRGIYMNAKVAF**C**IHNIAYQGRFAFSDFSLLNLPDEYKSSF
DFIDGYEKPVKGRKINWMKAGILESHRVVTVSPHYAQELVSGVDKGVELDNVLRKTCITGIVNGMDIQEWNPATDKYTDVNYDITTVMDAKPLLKEALQ
AAVGLPVDRKIPLIGFIGRLEEQKGSDILVAAIHKFIGLDVQIIVLGTGKKEFEQEIEQLEVLYPNKAKGVAKFNVPLAHMITAGADFMLVPSRFEP**C**GLIQL
HAMRYGTVPICASTGGLVDTVKEGYTGFHMGAFSVE**C**DVVDPADVLKIVTTVARALEIYGTLAFAEMIKN**C**MSQELSWKEPAKKWETLLLSLGAAGSEA
GVEGDEIAPLAKENVATP

>A0A1S4DL03_50S ribosomal protein L18
MQGTRLELEPQGSRTRTIQPINFRQFPRSTQSTSYPKVHLRTKKAMACTSLSLSFLHNACADNKQLTLSFRTKLVTSARPLTVEAKATTRREDRTARHV
RIRKKVEGTPERPRLCVFRSNKHIYVQVIDDSKMHTLASASTMQKPISEEFDYSAGPTTDVAKKVGEVIAKA**C**LEKGITKVAFDRGGYPYHGRIEALADA
AREHGLQF

NTRC targets

>A0A140G1P8_ATP synthase CF0 B subunit
MKNVTDSFVSLGHWPSAGSFGFNTDILATNPINLSVVLGVLIFFGKGVLSDLLDNRKQRILNTIRNSEELRGGAIEQLEKARSRLRKVESEAEQFRVNGY
SEIEREKLNLINSTYKTLEQLENYKNETIQFEQQRAINQVRQRVFQQALRGALGTLNS**C**LNNELHLRTISANIGMLGTMKEITD

>A0A1S3XVT6_Cytochrome b6
MATQTVENSSRSGPRRTAVGDLLKPLNSEYGKVAPGWGTTPLMGVAMALFAFDRGISLFLYFRTVRDESLIYGSQRGSSAYLNKVYDWFEERLEIQAIA
DDITSKYVPPHVNIFY**C**LGGITLT**C**FLVQVATGFAMTFYYRPTVTEAFASVQYIMTEANFGWLIRSVHRWSASMMVLMMILHVFRVYLTGGFKKPRELTW
VTGVVLAVLTASFGVTGYSLPWDQVGYWAVKIVTGVPDAIPVIGSPLVELLRGSASVGQSTLTRFYSLHTFVLPLLTAVFMLMHFPMIRKQGISGPL

>A0A1S4B832_Cytochrome b6-f complex iron-sulfur subunit
MASSTLSPVTQLCSSKSGLSSVSQCLLLKPMKINSHGLGKDKRMKVKCMATSIPADDRVPDMEKRNLMNLLLLGALSLPTAGMLVPYATFFAPPGSGG
GSGGTPAKDALGNDVIASEWLKTHPPGNRTLTQGLKGDPTYLVVENDGTLATYGINAV**C**THLG**C**VVPFNAAENKFI**C**P**C**HGSQYNNQGRVVRGPAPL
SLALAHADIDDGKVVFVPWVETDFRTGEAPWWA

>A0A140G1S8_Cytochrome f
MQTRNAFSWLKKQITRSISVSLMIYILTRTSISSAYPIFAQQGYENPREATGRIV**C**AN**C**HLANKPVEIEVPQAVLPDTVFEAVVRIPYDMQLKQVLANGKR
GGLNVGAVLILPEGFELAPPDRISPEMKEKIGNLSFQSYRPNKKNILVIGPVPGQKYSEITFPILSPDPATKKDVHFLKYPIYVGGNRGRGQIYPDGSKSN
NTVYNATAAGIVSKIIRKEKGGYEITITDASDGRQVVDIIPPGPELLVSEGESIKFDQPLTSNPNVGGFGQGDAEIVLQDPLRVQGLLFFLASVILAQIFLVLK
KKQFEKVQLAEMNF

>A0A140G1X0_Photosystem I iron-sulfur center
MSHSVKIYDT**C**IG**C**TQ**C**VRA**C**PTDVLEMIPWDG**C**KAKQIASAPRTED**C**VG**C**KR**C**ESA**C**PTDFLSVRVYLWHETTRSMGLAY

>A0A140G1R3_Photosystem I P700 chlorophyll a apoprotein A1
MIIRSPEPEVKILVDRDPVKTSFEEWARPGHFSRTIAKGPDTTTWIWNLHADAHDFDSHTSDLEEISRKVFSAHFGQLSIIFLWLSGMYFHGARFSNYEA
WLSDPTHIGPSAQVVWPIVGQEILNGDVGGGFRGIQITSGFFQIWRASGITSELQLY**C**TAIGALVFAALMLFAGWFHYHKAAPKLAWFQDVESMLNHHL
AGLLGLGSLSWAGHQVHVSLPINQFLNAGVDPKEIPLPHEFILNRDLLAQLYPSFAEGATPFFTLNWSKYADFLTFRGGLDPVTGGLWLTDIAHHHLAIAI
LFLIAGHMYRTNWGIGHGLKDILEAHKGPFTGQGHKGLYEILTTSWHAQLSLNLAMLGSLTIVVAHHMYSMPPYPYLATDYGTQLSLFTHHMWIGGFLIV
GAAAHAAIFMVRDYDPTTRYNDLLDRVLRHRDAIISHLNWA**C**IFLGFHSFGLYIHNDTMSALGRPQDMFSDTAIQLQPVFAQWIQNTHALAPGATAPGAT
ASTSLTWGGGDLVAVGGKVALLPIPLGTADFLVHHIHAFTIHVTVLILLKGVLFARSSRLIPDKANLGFRFP**C**DGPGRGGT**C**QVSAWDHVFLGLFWMYN
AISVVIFHFSWKMQSDVWGSVSDQGVVTHITGGNFAQSSITINGWLRDFLWAQASQVIQSYGSSLSAYGLFFLGAHFVWAFSLMFLFSGRGYWQELIE
SIVWAHNKLKVAPATQPRALSIIQGRAVGVTHYLLGGIATTWAFFLARIIAVG

>A0A140G1R2_Photosystem I P700 chlorophyll a apoprotein A2
MALRFPRFSQGLAQDPTTRRIWFGIATAHDFESHDDITEERLYQNIFASHFGQLAIIFLWTSGNLFHVAWQGNFESWVQDPLHVRPIAHAIWDPHFGQP
AVEAFTRGGALGPVNIAYSGVYQWWYTIGLRTNEDLYTGALFLLFLSAISLIAGWLHLQPKWKPSVSWFKNAESRLNHHLSGLFGVSSAWTGHLVHVAI
PASRGEYVRWNNFLDVLPHPQGLGPLFTGQWNLYAQNPDSSSHLFGTAQGAGTAILTLLGGFHPQTQSLWLTDIAHHHLAIAFIFLVAGHMYRTNFGIG
HSMKDLLDAHIPPGGRLGRGHKGLYDTINNSLHFQLGLALASLGVITSLVAQHMYSLPAYAFIAQDFTTQAALYTHHQYAGFIMTGAFAHGAIFFIRDYNP
EQNEDNVLARMLEHKEAIISHLSWASLFLGFHTLGLYVHNDVMLAFGTPEKQILIEPIFAQWIQSAHGKTSYGFDVLLSSTSGPAFNAGRSIWLPGWLNA
VNENSNSLFLTIGPGDFLVHHAIALGLHTTTLILVKGALDARGSKLMPDKKDFGYSFP**C**DGPGRGGT**C**DISAWDAFYLAVFWMLNTIGWVTFYWHWKHI
TLWQGNVSQFNESSTYLMGWLRDYLWLNSSQLINGYNPFGMNSLSVWAWMFLFGHLVWATGFMFLISWRGYWQELIETLAWAHERTPLANLIRWRD
KPVALSIVQARLVGLAHFSVGYIFTYAAFLIASTSGKFG

>A0A1S3ZIE1_Photosystem I reaction center subunit II
MAMATQASLFTPALSAPKSSAPWKQSLASFSPKQLKSTVSAPRPIRAMAEEAATKEAEAPVGFTPPQLDPNTPSPIFGGSTGGLLRKAQVEEFYVITWE
SPKEQIFEMPTGGAAIMREGANLLKLARKEQ**C**LALGTRLRSKYKINYRFYRVFPNGEVQYLHPKDGVYPEKVNAGRQGVGQNFRSIGKNKSPIEVKFTG
KQVYDL

>A0A1S4BQS3_ Photosystem I reaction center subunit XI
MATAASTTMASQLKSSFASSLTRGNGLVTPKGISGAPFKIFPSTRKSCFTIKAVQTDKPTYQVIQPLNGDPFIGSLETPVTSSPLIAWYLSNLPAYRTAVN
PLLRGVEVGLAHGFLLVGPFVKTGPLRNTEYAGGAGSLAAAGLVVILSI**C**LTIYGISSFKEGEASTAPALTLTGRKKVPDQLQTAEGWSKFTGGFFFGGIS
GVTWAYFLLYVLDLPYYVK

>A0A1S4AH01_10 kDa chaperonin-like
MASTFIAVAKPFTSHSTNLTSFSTQRPIGLKRNSLRINAISKKWEPTKVVPQADRVLIRLEELSEKSAGGVLLPKSAVKFERYLMGEVLSVGSEVAQVEAG
KKVLFSDINAYEVDLGTDARH**C**F**C**KESELLALVE

>A0A1S3ZRR1_Nucleoid-associated protein At4g30620
MASTSALSAGISNLHNFSSVSFYNPSPNVNQVGMWTLSRSGCRKVSDNPRPMQIRALFGGKKDDNENNKAGLLGNMQNLYETVKKAQNVVQVEAVR
VQKELALAEFDGY**C**EGELIKVTLSGNQQPIRTEITEAAMELGPEKLSLLITEAYKDAHQKSVLAMKERMSDLAQSLGMPAGLGEGFKQ

>A0A1S4CGA5_Pentatricopeptide repeat-containing protein At4g30825
MASLKLSFYVDKSWESKKLKFNVKALNFTDSKCLVPSFLGYGYVGGAFVVNPFCNLKHIRVSRLETEELETSELSLLDGERVDNFEGDLGNESLVSERL
NLGGVSQKGKFNVWKRFRRVKRVVNNSKYRSSFREKDRNHGMQEKTKIVFDEISEENVIGSLNGVDFDVGNIGSDSSLEH**C**NAILKQLESGDDGKALS
FFRWMQKNGKLKQNVTAYNLILRVLGRRGDWDGAEAMIKEMSLESGCELTYQVFNTLIYACHKKGLVELGAKWFHMMLENRIQPNIATFGMLMALYQK
GWNVEEAEFTFSKMRSLKIM**C**QSAYSAMLTIYTRMRLYDKAEKIIGFLREDEVILNQENWLVLLNAYCQQGKLAEAEQVLASMKQSGFSPNIVAYNTLIT
GYGKISNMRAAQRLFSDLERVGMEPDETTYRSMIEGWGRADNYEEARRYYVELKRLGHKPNSSNLYTMLNLQVKHGDEEDVVSTVEEMMHSGSEKS
TVLGILLQAYEKLECVHKVPSILRGSLYDHVLRNQISCSSLVMAYVENSMIDDALKVLREKRWEDALFEDNLYHLLI**C**S**C**KDLGYPENAVKVFACMPKSY
KPNLHIICTMIDIYSTINDFAEAEKLYLMLKNSDVKLDMITLSVVVRMYVKSGALEEA**C**SVLDAMEKQKNIVPDTYLLRDMLRIYQR**C**DKQDKLADLYYKLV
KRGVIWDQEMYS**C**VIN**CC**ARALPVDELSRLFDEMLKHGFLPNTVTFNVMLDVYGKSRLFKRAREVFSMAKKRGLADVISYNTLIAAYGRSKDFKNMSST
VKKMHFNGFSVSLEAYNCMLDAYGKEGQMEKFRSILQRLKESGHSSDHYTYNIMINIYGELGWIEEVANVLTELKESGIGPDLCSYNTLIKAYGIAGMVE
SAADLVKEMRKNGIEPDRVTYANLINALRKNDMFLEAVKWSLWMKQIGL

>A0A1S4C5X4_Oxygen-dependent coproporphyrinogen-III oxidase
MLTPILSSASCSWTPTSQFPHSWHSSPSFLTKPLNLPFTVSYKTAKKPTPNYSFKVQAMIEKEVAESHKPDTFLRESDMGSNVTSNSSSVRGRFEKMIR
EAQDSVCLAIEKADGGAKFKEDVWSRPGGGGGISRVLQDGAVFEKAGVNVSVVYGVMPPEAYRAARPTDNGNVKPGPIPFFAAGVSSVLHPKNPFAP
TLHFNYRYFETDAPKDAPGAPRQWWFGGGTDFTPAYIFEEDVKHFHSVQKAA**C**DKFDASFYPRFKKW**C**DDYFYIKHRDERRGLGGIFFDDLNDYDQE
MLLSFSTECANSVIPAYIPIIEKRKDTPFTDKHKAWQQLRRGRYVEFNLVYDRGTTFGLKTGGRIESILVSLPLTARWEYDHKPEEGTEEWKLLDACINPK
EWI

>A0A1S3X073_Phosphoglycolate phosphatase 1B
MLSSRVTAAISSSTTATFLFNNPKISSKKFPYISNPLNNSAKSIKWNCRNSRMEKSASSFVTKASAQPLTNPGELIDSVETFIFD**C**DGVIWKGDKLIDGVPE
TLDLLREKGKRLVFVTNNSTKSRKQYGKKFETLGLSVSEEEIFASSFAAAAYLKSIDFPKDKKVYVVGEEGILKELELAGIQHIGGPEDGDKKIELKPGYM
MEQDKDVGAVVVGFDRYFNYHKIHKLHC**C**CSVASGGGSMVGAILGSTKREPLVVGKPSTFMMDYLANEFNIQKSQI**C**MVGDRLDTDILFGQNGG**C**KTL
LVLSGVTSLSMLQDPKNSIQPDFYANKISDFLSIKAAAV
